# Host Genetics, Innate Immune Responses, and Cellular Death Pathways in Poliomyelitis Patients

**DOI:** 10.3389/fmicb.2019.01495

**Published:** 2019-07-09

**Authors:** Nanna-Sophie B. Andersen, Simon M. Larsen, Sara K. Nissen, Sofie E. Jørgensen, Maibritt Mardahl, Mette Christiansen, Lise Kay, Trine H. Mogensen

**Affiliations:** ^1^Department of Infectious Diseases, Aarhus University Hospital, Aarhus, Denmark; ^2^Department of Biomedicine, Aarhus University, Aarhus, Denmark; ^3^Department of Clinical Immunology, Aarhus University Hospital, Aarhus, Denmark; ^4^Specialized Hospital for Polio- and Accident Patients, Rødovre, Denmark; ^5^Department of Clinical Medicine, Aarhus University, Aarhus, Denmark

**Keywords:** poliovirus, paralytic poliomyelitis, innate immunity, interferon, autophagy, apoptosis, whole exome sequencing

## Abstract

**Purpose:**

Poliovirus (PV) is one of the most studied viruses. Despite efforts to understand PV infection within the host, fundamental questions remain unanswered. These include the mechanisms determining the progression to viremia, the pathogenesis of neuronal infection and paralysis in only a minority of patients. Because of the rare disease phenotype of paralytic poliomyelitis (PPM), we hypothesize that a genetic etiology may contribute to the disease course and outcome.

**Methods:**

We used whole-exome sequencing (WES) to investigate the genetic profile of 18 patients with PPM. Functional analyses were performed on peripheral blood mononuclear cells (PBMCs) and monocyte-derived macrophages (MdMs).

**Results:**

We identified rare variants in host genes involved in interferon signaling, viral replication, apoptosis, and autophagy. Upon PV infection of MdMs, we observed a tendency toward increased viral burden in patients compared to controls, suggesting reduced control of PV infection. In MdMs from patients, the IFNβ response correlated with the viral burden.

**Conclusion:**

We suggest that genetic variants in innate immune defenses and cell death pathways contribute to the clinical presentation of PV infection. Importantly, this study is the first to uncover the genetic profile in patients with PPM combined with investigations of immune responses and viral burden in primary cells.

## Introduction

Paralytic poliomyelitis (PPM) is a rare disease presentation following poliovirus (PV) infection. In 1988, The global polio eradication initiative (GPEI) launched a program in which global expansion of the PV vaccine resulted in reduction of PPM from an annual level of at least 350,000 cases to only 22 cases caused by wild poliovirus (WPV) in 2017. These cases were identified in the two endemic countries Afghanistan and Pakistan with 14 and 8 cases, respectively (Khan et al., 2017). In 2017, circulating vaccine-derived poliovirus (cVDPV) paralyzed 22 and 74 children in the Democratic Republic of Congo and Syria, respectively. In 2018, the first case of PPM in Papua New Guinea in 18 years was diagnosed, and the total number of cases of WPV and cVDPV reached 33 and 104, respectively. To date, in 2019, the year-to-date of WPV is almost twice the size of the year-to-date in 2018 (The Global Polio Eradication Initiative [GPEI], 2018). Importantly, every time 1–2 cases of PPM caused by WPV type I are reported, an estimated 100 individuals have been infected (Nathanson and Martin, 1979; Nathanson and Kew, 2010). The failing achievement of stopping PV transmission challenges global eradication.

Poliovirus spreads by the fecal–oral route, and the majority of infections follow a benign disease course. In a minority of cases, estimated to 0.1–1% of infected individuals, the infection leads to permanent paralysis or death due to infection of motor neurons in the central nervous system (CNS) (Pfeiffer, 2010). Knowledge of disease determinants has remained sparse (Bodian, 1955; Sabin, 1956; Nathanson, 2008), and the invasion of the CNS has been described as accidental (Mueller et al., 2005; Racaniello, 2006). PV is a single stranded RNA virus belonging to the picornavirus family with sparse knowledge on the cellular and humoral determinants of protective immunity in humans (Nathanson, 2008). However, a recent study in a non-human primate PPM model demonstrated that early in the infectious process, PV replication occurs in both epithelial cells, explaining virus shedding in the gastrointestinal tract, and lymphoid/monocytic cells in tonsils and Peyer’s patches, explaining subsequent viremia, extending previous studies of PV pathogenesis in humans (Shen et al., 2017). It is important to acknowledge that circulating antibodies have a protective effect from disease (Lennette and Schmidt, 1957). However, studies in mice and cell cultures have suggested that innate immune responses, including type I interferon (IFN), apoptosis, and autophagy, play a role in the control of PV. Studies in PV receptor (PVR) transgenic (tg) mice revealed the difficult challenges in identifying the exact mechanisms of how PV signals within infected cells. The Toll like receptor 3 (TLR3) pathway as well as the melanoma differentiation-associated protein 5 (MDA5) pathway are suggested to be mandatory for antiviral PV signaling (Oshiumi et al., 2011; Abe et al., 2012). Furthermore PVRtg IFNα/β receptor (IFNAR) knockout mice cannot control PV *in vivo*, and type I IFN is believed to be important in control of the viremia in non-neural tissue (Ida-Hosonuma et al., 2005). Autophagy is a complex mechanism originally discovered as a stress- and starvation response to maintain cellular homeostasis (Levine and Kroemer, 2008). Within the past years, an antimicrobial role against pathogens, including PV, has been linked to autophagy (Levine, 2005; Deretic and Levine, 2009; Delorme-Axford et al., 2013; Staring et al., 2017). Importantly, autophagy plays a pivotal role in maintaining cellular homeostasis and preventing degeneration in cells of the CNS, including the motor neurons, which have limited regenerative capacity compared to immune cells (Gonzalez Porras et al., 2018; Stavoe and Holzbaur, 2018). Finally, enteroviruses possess several mechanism of delaying cell death to antagonize the fact that the viral infection induces cell death pathways. Apoptosis is one such cell death mechanism, and control of the cellular homeostasis of apoptosis is crucial for PV replication as well as host cell survival and viral restriction (Tolskaya et al., 1995; Harris and Coyne, 2014).

These described mechanisms might contribute to immune control of PV prior to achievement of long-term immunity. Following a natural infection or vaccination with the oral sabin vaccine (OPV) and/or the inactivated Salk vaccine (IPV), a protective neutralizing antibody response is mounted (Dotzauer and Kraemer, 2012). The adaptive T-cell responses and the role of these cells in the resolution of PV infection are less well-described, thus virus-specific CD4+ and cytotoxic CD8+ T-cell responses, and long-term memory cells might play a role in protective immunity (Wahid et al., 2005b).

The OPV became the backbone of worldwide vaccine strategies launched by GPEI. However, OPV immunization has been demonstrated to cause infection, paralysis, and continuous shedding of neurovirulent VDPV in some patients with primary immunodeficiencies (PIDs) (Shaghaghi et al., 1995; Guo et al., 2015). The GPEI plans to cease use of OPV once circulating PV has been eradicated. However, there are no means for addressing the threat posed by existing PID patients infected with VDPVs, neither to the individual risk of PPM, nor to the community in terms of having a continuous source of PV shedding (Global Polio Eradication Initiative [GPEI], 2013, 2015; Garg et al., 2018). This highlights the relevance of the current study for focus on PV pathogenesis and immunity and the importance of inclusion of PID patients in PV surveillance programs.

It is generally accepted that single-gene inborn errors of innate immunity are associated with enhanced susceptibility to infections (Casanova et al., 2011; Sancho-Shimizu et al., 2011; Andersen et al., 2015; Ogunjimi et al., 2017; Jorgensen et al., 2018). However, there are no studies on human PV infections examining the role of genetic predisposition underlying PV pathogenesis. In the present work, we identified 18 patients with PPM contracting PV in early childhood, and performed whole-exome sequencing (WES) together with functional studies on patient PBMCs and MdMs. To our knowledge, this is the first study to uncover the genetic profile of patients with PPM. Here we describe rare variants in genes predicted to influence viral sensing, and replication, host cellular autophagy and apoptosis, as well as acetylcholine receptor biology.

## Materials and Methods

### Study Population

The patient group consisted of 11 males and seven females of Caucasian origin (49–81 years, median age 71 years) and with a diagnosis of PPM based on clinical presentation in the pre-vaccination years (1940–1950) or for the one patient of Asian origin (who was adopted following infection in her country of origin), infected in the early 1970s (Supplementary Table S1). We considered a severe phenotype as complete or partial paralysis in several parts of the body or respiratory muscles, or complete paralysis in one limp. A total of 18 patients were invited and enrolled in cooperation with Polio Denmark (The Danish National Polio association), which has contact information of 100 PPM patients. Fourteen healthy gender-matched controls were recruited from Aarhus University Blood bank. The controls were of Caucasian origin with an age range of 23–60 years (median age 33 years). All of the controls were immunized against PV.

### WES and Bioinformatics

**FIGURE 1:**
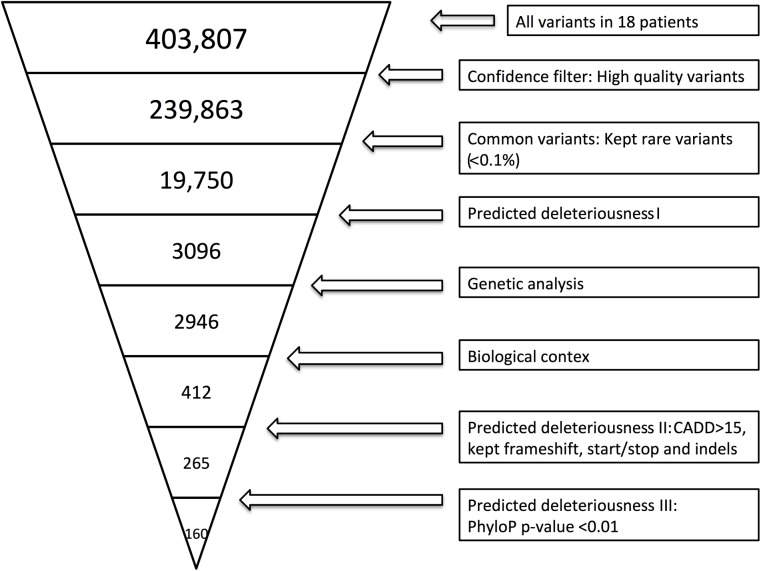
Whole-exome sequencing (WES) filtering diagram. In brief, we included exonic variants that were predicted to be rare (present in <0.1% of the reference genomes), excluded variants with CADD score <15 (Kircher et al., 2014), and included variants with frameshift, in-frame insertions or deletions (indels) or stop codon change (No CADD score available for these types of variants). Lastly, we included genes with high evolutionary conservation with a phyloP *p*-value less or equal to 0.01. The variant filtering was verified by random sampling, and all BAM files of the variants identified in Ingenuity Variant Analysis (IVA) were manually examined in order to include only variants with high sequencing quality. BAM files were evaluated by use of the UCSC genome browser or IGV.

Whole-exome sequencing was performed as previously described (Nissen et al., 2018). Variant call files (VCF) were filtered using the approach described in Supplementary Method 1. Figure 1 illustrates the filtering process. To identify the specificity of variants found within the cohort, we used the same filter settings in a cohort of HSE patients.

The bioinformatics include two steps: evaluation of the identified variants, and evaluation of the variant-containing genes. First, following the identification of variants by Ingenuity, we included additional tools in order to eliminate variants not contributing to the phenotype. The SIFT (Kumar et al., 2009) and PolyPhen-2 (Adzhubei et al., 2010; Andersen et al., 2017) score were extracted from the IVA analysis. The PolyPhen-2 score predicts the possible impact of an amino acid substitution on the structure and function of a human protein. This score represents the probability that a substitution is damaging (Table 1). A SIFT score predicts whether an amino acid substitution affects protein function. The SIFT score ranges from 0.0 (deleterious) to 1.0 (tolerated) (Supplementary Table S3). Second, the Mutation Significance Cut-off (MSC) (with a 99% confidence Interval with HGMD Database Source) (Belov et al., 2003) was calculated for each variant-containing gene in order to include genes with a high phenotypic effect (possibly damaging) defined as any combined annotation dependent depletion (CADD) score greater than the MSC. The importance of this quantitative approach with gene-level and gene-specific cut-off values improves the use of variant-level methods, CADD in particular. Third, in order to estimate genetic intolerance, variant-containing genes were included based on function, ExAC RVIS (Residual Variation Intolerance Score based on exome aggregation consortium data) (Petrovski et al., 2013), gene damaging index (GDI) (Itan et al., 2015), ExAC missense *Z* score and LoF PLI (Lek et al., 2016; Supplementary Table S3). We excluded genes with 3 or more scores that did not predict the variant to be disease-causing or the gene to be intolerant, unless the remaining values including the function were highly relevant to the phenotype of PPM.

**Table 1:** Rare variants identified in patients with paralytic poliomyelitis.

ID	Gene	Gene function	Transcript variant	Protein variant	CADD	MSC	PP-2	GnomAD frequency (%)	Cat.
1	*CREBBP*	Activation of IRF	c.5770G > A	p.V1924M	21.4	0,28	B	0.034	1
	*VPS16*	Protein transport to lysosomes	c.140T > C	p.I47T	23.2	5.442	PoD	0.001	3
2	*CC2D1A*	IFNβ promoter activation	c.1345G > A	p.V449M	19.74	3.313	B	0.06	1
	*TP53BP2*	Interaction with BCL2	c.1652A > T	p.K551I	17.34	3.313	PoD		4
3	*ERAP1*	Aminopeptidase, trims peptide for MHC I	c.1811T > C	p.M604T	23.6	3.313	PoD		6
	*MX1*	ISG	c.437C > A	p.A146D	27.4	3.313	PoD	0.003	2
	*TNIP1*	Inhibition of NFkB	c.537G > T	p.Q179H	28.3	3.313	PrD		1,2
4	*DHX36*	dsRNA sensing	c.1678A > G	p.I560V	27	3.313	PrD		1
	*ULK1*	Initiation of autophagy	c.1096+1G > A	SSL	25.8	3.313			3
6	*NOS2*	Reactive free radical	c.1141C > T	p.R381W	35	3.313	PrD	0.003	2
	*BNIP2*	Binds BCL2	c.904C > A	p.P302T	28.1	3.313	B		4
	*CHRNA1*	Acethylcholine receptor subunit, muscle	c.224G > A	p.R75H	35	16.23	PrD	0.045	5
7	*UBA7*	Target abnormal protein for degradation	c.2864C > T	p.A955V	23.3	3.313	B	0.01	2
	*CTSL*	Lysosomal proteinase	c.205G > A	p.G69R	25.9	3.313	PrD	0.002	3
	*CTSC*	Lysosomal proteinase	c.1324C > T	p.R442C	29.2	9.015	B	0.013	3
	*FOS*	Involved in AP-I transcription factor formation	c.467A > G	p.N156S	25.3	3.313	PrD		1
8	*ANXA6*	Involved in endo/exocytosis	c.236C > T	p.T79M	19.91	5.522	B	0.042	2
	*MMP8*	Metallopeptidase	c.1225+1G > A	SSL	23.3	3.313		0.067	6
9	*GBP1*	Hydrolyses GTP to GMP, ISG	c.1352C > T	p.P451L	26.1	3.313	PrD	0.009	2
	*CHRNA7*	Acethylcholine receptor subunit, neuronal	c.431G > C	p.G144A	22.5	3.313	PoD	0.002	5
	*USP25*	Stabilization of TRAF3 and TRAF6	c.2489C > T	p.A830V	21.9	3.313	PrD	0.001	1
10	*ULK1*	Initiation of autophagy	c.700G > A	p.E234K	34	3.313	B		3
	*ANXA5*	Binding of phosphatedylserine, apoptosis	c.278C > T	p.A93V	23.5	3.313	PoD		2, 4
	*ARHGAP21*	Regulation of IAV NA transport	c.3154G > A	p.D1052N	31	3.313	PrD		2
11	*BNIP3*	Interaction with BNIP2	c.773C > A	p.T258N	26.7	14.52	B		4
12	*TRAF2*	NFkB activation, anti-apoptotic	c.1462G > A	p.D488N	29.9	3.313	PrD	0.017	1, 2, 4
	*PTPN22*	Bindsing ofTRAF3, regulation of IFN type I	c.1865C > G	p.P622R	27.4	3.313	PrD	0.018	1
	*NOD1*	Bacterial and HCV dsRNA sensing	c.1271T > C	p.V424A	20.1	3.313	B		1
	*TP53BP2*	Interaction with BCL2	c.2770C > T	p.H924Y	26.2	3.313	PrD	0.012	4
15	*CHRNG*	Acethylcholine receptor subunit, fetal, muscle	c.1421G > A	p.R474H	34	0,298	PoD	0.008	5
	*PMAIP1*	Pro-apoptotic	c.3G > T	p.M1I	22.3	NA	PrD	0.003	4
	*VPS18*	Protein transport to lysosomes	c.1757T > C	p.L586P	23.6	3.313	PrD	0.009	3
16	*ATG7*	Involvement in autophagy, axonal homeostasis	c.1162G > A	p.A388T	33	3.313	B	0.02	3
	*CHRNA5*	Acethylcholine receptor subunit, neuronal	c.226T > G	p.F76V	25.7	3.313	PrD	0.018	5
17	*ANXA6*	Involvement in endo/exocytose	c.1060A > C	p.I354L	24.7	5.522	PrD		2
	*TRIM67*	Increasing innate immune responses	c.961G > A	p.G321R	25.9	3.313	PoD	0.002	1
18	*C3AR1*	Involvement in complement	c.209T > C	p.L70P	26.7	3.313	PrD	0.002	6
	*CHRNA10*	Acethylcholine receptor subunit, neuronal	c.207+2T > G	SSL	24.7	3.313		0.013	5
	*MMP2*	Metallopeptidase	c.1573G > A	p.E525K	34	2.28	PrD	0.001	6


*Variants identified in patients with PPM. *ID*, patient number; *Cat*, categorization of gene function. 1, Intracellular signaling; 2, viral replication; 3, autophagy; 4, apoptosis; 5, acethylcholine receptor family; 6, no uniform classification. *PP-2*, PolyPhen-2: The PolyPhen-2 score ranges from 0.0 (tolerated/benign) to 1.0 (deleterious/damaging). Variants with scores of 0.0 are predicted to be benign. Values closer to 1.0 are more confidently predicted to be deleterious. The score can be interpreted as follows: 0.0 to 0.15, variants with scores in this range are predicted to be benign. 0.15 to 1.0, variants with scores in this range are possibly damaging. 0.85 to 1.0, variants with scores in this range are more confidently predicted to be damaging (probably damaging). *Pr.D*, probably damaging; *Po.D*, possibly damaging; *B*, Benign; *CADD*, combined annotation dependent depletion score; *MSC*, mutation significance cut-off score; *GnomAD*, genome aggregation database; *IRF*, interferon regulatory factor; *IFNβ*, interferon beta; *MHC*, major histocombability complex: *ISG*, interferon stimulated gene; *NFκB*, nuclear factor kappa B; *dsRNA*, double stranded RNA; *GTP*, guanosinetriphosphate; *GMP*, guanosinmonophosphat; *TRAF*, tumor necrosis factor receptor-associated factor; *IAV*, influenza A virus; *NA*, neuraminidase A; *HCV*, hepatitis C virus; *SSL*, splice site loss. PolyPhen-2 predicts the possible impact of amino acid substitutions. Therefore, the algorithm cannot calculate a value for splice site loss. See Supplementary Methods 2 for detailed description of the variant-containing genes.*

In addition, we performed an independent second layer of analysis by searching for single nucleotide polymorphisms (SNPs) in *IFNAR1*, *TLR3*, and *IFIH1*, which have been associated with increased susceptibility to enterovirus infections. In this analysis we did not apply a filtration pipeline, except for a biological filter including *IFNAR1, TLR3* and *IFIH1*.

### STRING Analysis

The 36 variant-containing genes were analyzed for associations regarding physical interactions or shared functionality using©STRING consortium 2017, version 10.5, creating an association network based on data from genomic context predictions, high-throughput experiments, co-expressions, automated textmining, and database search (Szklarczyk et al., 2017).

### Patient Material

Blood was collected in Lithium Heparin-stabilized tubes and isolated as previously described (Nissen et al., 2018). MdMs were differentiated from PBMCs as previously described (Jorgensen et al., 2018).

### Poliovirus Infection

Infections were performed with Human PV 1, LSa strain (a variant of the Mahoney strain), (ATCC^®^VR-59^TM^ Mannassas, VA, United States) at 100 MOI for PBMCs or 10 MOI for MdMs. Infections were carried out as previously described (Jorgensen et al., 2018).

### Total RNA Isolation, cDNA Synthesis, and RT-qPCR

RNA isolation, cDNA synthesis, and RT-qPCR were carried out as previously described (Carter-Timofte et al., 2018; Jorgensen et al., 2018; Nissen et al., 2018) using the following primers and probes (gene, catalog nr., assay ID) all from Thermo Fischer Scientific: TBP (4331182, Hs00427620_m1), CXCL10 (4331182, Hs01124251_g1), TNFα (4331182, Hs01113624_g1), IFNB1 hCG28967 (4331182 Hs01077958_s1), IL6 hCG38231 (4331182 Hs00985639_m1), and 18S (4331182, Hs03928985_g1).

### Viral Replication in MdMs

We used a modified method previously described (Wahid et al., 2005a). Briefly, MdMs were stimulated with PV at 10 MOI for 1 h, and MdMs were washed 7 times before adding fresh media. The supernatants were harvested at 24 h post infection. The supernatants were applied in serial dilution to a confluent monolayer of HeLa cells. The viral titer was measured by end-point dilution assay and calculated by the Reed-Munch method.

### Mesoscale

Peripheral blood mononuclear cells were seeded for overnight incubation at a concentration of 5 × 10^5^ cells/mL in RPMI supplemented with 10% FCS and 1% P/S. The cells were infected with PV at an MOI of 100 or stimulated with 100 ug/mL high molecular weight (HMW) Poly(I:C) (tlrl-pic, Invivogen). Following 24 h of incubation, IFNs were measured in cell culture supernatants using U-PLEX Interferon Combo Human (Mesoscale Diagnostics, Catalog number K15094K-2, Lot number 285545) on a Meso Quickplex SQ 120 instrument according to the manufacturer’s instructions.

### Statistics

Experiments were performed in experimental duplicates or triplicates. Differences between patient and controls were calculated using non-parametric Mann-Whitney test (Graphpad Prism 6). Correlation analyses were performed by Spearman correlation.

### Ethics Statement

The project was approved by the Danish National Committee in Health Research Ethics (#1-10-72-66-16) and the Danish Data protection Agency (#1-16-02-216-16) in accordance with the ethical standards of the Helsinki Declaration. Following oral and written information, all patients provided written consent prior to inclusion. The part of the project involving WES analysis of the HSE cohort, was approved by Danish National Committee in Health Research Ethics (#1-10-72-275-15) and previously published (Mork et al., 2015).

## Results

### Identification of Rare Genetic Variants in Patients With Paralytic Poliomyelitis

**FIGURE 2:**
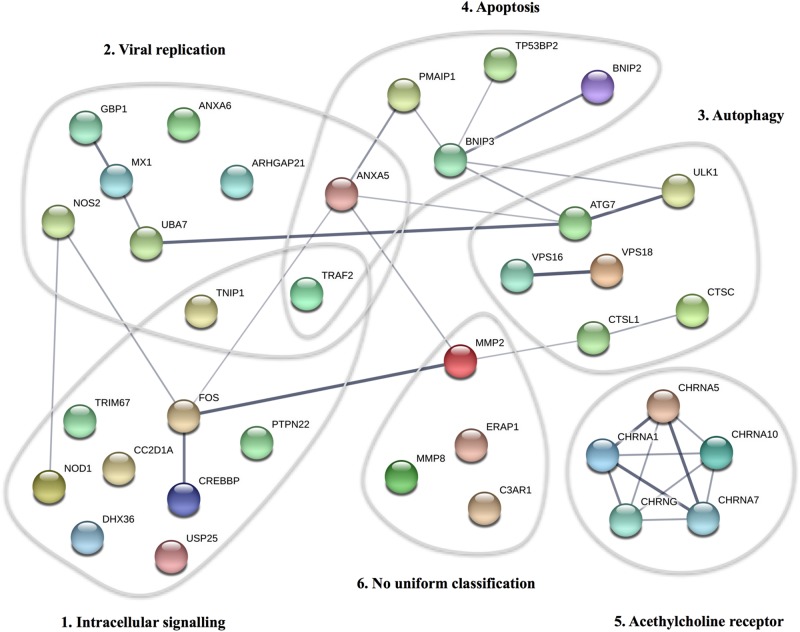
STRING protein-protein interaction network for proteins described in the PPM cohort. Each circle represents the protein affected by at least one variant in the cohort. The thickness of the gray lines represents strength of data supporting a protein-protein interaction. The gray circles include clusters of proteins involved in common pathways/biological functions. The PPI enrichment *p*-value for the number of identified edges (30) compared to expected (9) in a group of 36 proteins was 4.13 × 10^-8^, thus significantly more than expected, with a minimum interaction score a 0.4.

By WES a total of 403,827 variants were identified in 20,789 genes. Following the ingenuity variant analysis (IVA) filtering described in Figure 1, the section “Materials and Methods” and Supplementary Method 1, we sorted out 160 variants in 163 genes. In order to identify false positive variants, we evaluated all variants and variant-containing genes using *in silico* tools (MSC, RVIS, GDI, ExAC missense *Z* score, LoF PLI, SIFT, and PolyPhen-2). Table 1 shows the final 39 variants in 36 genes, on which we decided to focus as potentially damaging and thus directly contributing to the phenotype (see Supplementary Method 2 and Supplementary Table S3 for a description of the variant-containing gene). All variants were heterozygous missense mutations, with the exception of P9 being homozygous for a variant in *CHRNA7*. None of the identified rare variants were shared between at least two patients in that same cohort, suggesting that the PPM phenotype might result from several genotypes. Notably, the majority of the variant-containing genes have similar biological functions or are part of common signaling pathways, suggesting a functional importance. To illustrate this point, the final list of 36 genes in the cohort is depicted in a STRING protein-protein interaction network based on close functional associations or physical interactions (Figure 2). The network had significantly (PPI enrichment *p*-value = 4.13.10^-8^) more interactions than expected (30 found vs. 9 expected) for a random set of 36 different proteins. This result was somehow expected as the 36 genes origin from a pre-selected list of genes (Supplementary Table S2). To illustrate to what extent the 36 genes included in the PPM cohort represented a genuine enrichment, we analyzed the PPI enrichment *p*-value 50 times in random sets of 36 proteins generated from the biological filter list (Supplementary Table S2). This analysis demonstrated that 66 vs. 34% of the analyzed random sets of proteins was found with a PPI enrichment *p*-value of non-significance vs. significance. This analysis, in combination with a significant *p*-value from the PPM cohort, indicates a degree of biological connection between the 36 genes in the PPM cohort. Noteworthily, the *p*-value from the PPM cohort network showed the strongest degree of significance.

In the independent additional analysis, we observed an interesting genotype in SNPs within *IFNAR1, TLR3*, and *IFIH1* (Supplementary Table S4). Starting at 403,807 variants, 22 variants were found in *IFIH1*, 11 variants were found in *TLR3*, and 15 variants were found in *IFNAR1*. Interestingly, we found an increased representation of 2 alleles in *IFIH1* (rs3747517 and rs1990760) as well as 1 allele in *TLR3* (rs3775291) compared to the frequency in GnomAD. These alleles have been reported to affect the host immune response to and/or disease outcomes of enterovirus infections (Gorbea et al., 2010; Cinek et al., 2012; Pang et al., 2014; Witso et al., 2015). The analysis revealed an interesting genetic composition of patients with PPM, lending further support for a role of the TLR3, and MDA5 pathways in anti PV immunity which should be further investigated in a larger cohort to confirm our observations.

### Identification of Genetic Variants in a Control Cohort of 18 Patients With Herpes Simplex Encephalitis (HSE)

In order to identify variants unique to the PPM phenotype, we performed IVA analysis using identical selection and filtering tools on a cohort of 18 HSE patients, who already had their genomes sequenced for research, and diagnostic purposes (Mork et al., 2015). Among 167 variants (Supplementary Table S5 lists the variant containing genes), 7 genes had non-identical variants in both cohorts. Only *ANXA5* and *C3AR1* were kept in the final list of the 39 variants reported in the present article. The 5 genes we excluded in the final list as it did not focus on PV infection or were excluded based on RVIS, GDI, ExAC missense *Z* score, LoF PLI, SIFT, and PolyPhen-2 as previously described. None of the patients reported any episode of HSE, and we had no reason to believe that the PPM patients suffered from a broader primary immune deficiency; hence we could not find a good argument to keep the variants located in genes present in both cohorts. We conclude that despite this minor overlap, the vast majority of variants identified were specific to the PPM cohort and of potential relevance for disease progression.

### Functional Innate Immune Responses and Viral Replication

**FIGURE 3:**
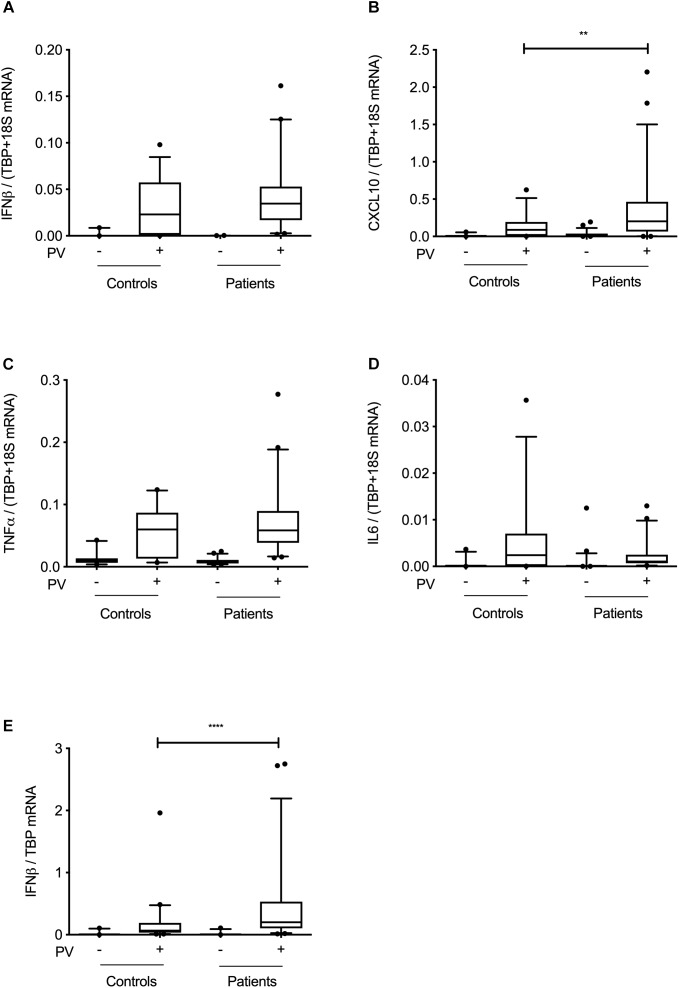
Innate immune responses at cohort level. PBMCs **(A–D)** or MdMs **(E)** from patients and controls were infected with PV at an MOI of 100 (PBMCs) or an MOI of 10 (MdMs). Total RNA was harvested 6 h following infection and subjected to RT-qPCR for measurement of IFNβ, CXCL10, TNFα, or IL6. Cytokine mRNA levels were normalized to the housekeeping gene TBP (MdMs) or TBP+18S (PBMCs) and compared to the pooled results of a total of nine controls. Data are shown as box plots with the 5–95% population, and outliers shown as independent dots. Non-parametric Mann-Whitney ranked sum test was used for statistical analysis. ^∗∗^*P* ≤ 0.01 and ^∗∗∗∗^*P* ≤ 0.0001. PV; poliovirus, PBMCs*;* peripheral blood mononuclear cells, MdMs; monocyte-derived macrophages.

**FIGURE 4:**
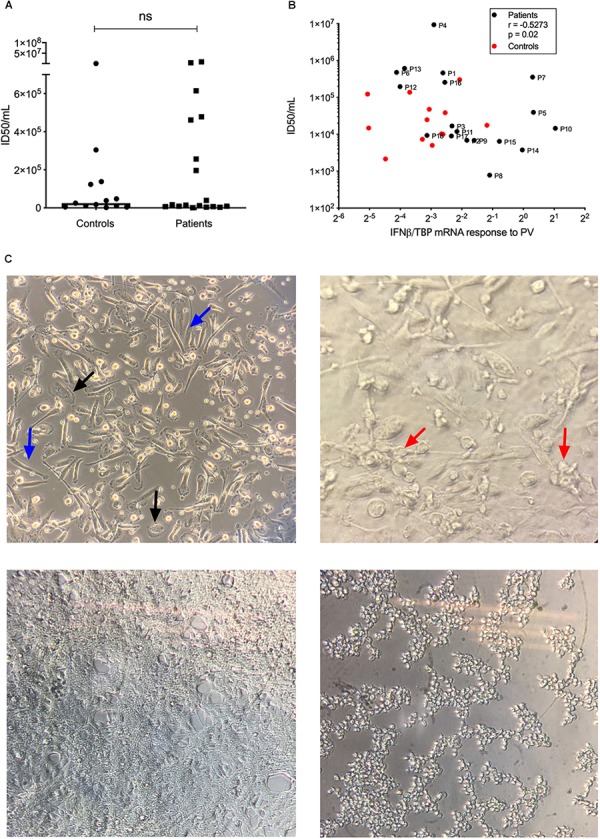
Viral replication in MdMs. MdMs were stimulated with PV at an MOI of 10 for 1 h. The inoculum was removed and cells were washed seven times before adding fresh media. The supernatants were harvested at 24 h (total infection time) following infection.**(A)** ID50/mL is shown as dots representing mean of triplicates or more from one or two independent experiments, box-line represent median. **(B)** Correlation of IFNβ mRNA response and ID50/mL. Non-parametric Mann-Whitney ranked sum test and Spearman correlation was used for statistical analysis. **(C)** Top left, uninfected MdMs. Black arrows, M1 morphology; blue arrows, M2 morphology. Top right, MdMs 24 h following infection. Red arrows, cells have started clustering and appear rounded as a sign of infection. Bottom left, a confluent monolayer of uninfected HeLa cells. Bottom right, infected HeLa cells. Cells have rounded and detached from the surface which indicate infection and/or cell death. PV, poliovirus; MdMs, monocyte-derived macrophages.

To functionally examine antiviral and proinflammatory innate immune responses to PV infection *in vitro*, we infected patient and control PBMCs and MdMs with PV and measured Type I IFN, CXCL10, and the proinflammatory cytokines TNFα and interleukin (IL)6. Compared to controls, PV infection of PBMCs induced a significantly enhanced CXCL10 mRNA response (Figure 3A–D). Despite differences at the individual level, no significant differences in IFNβ, TNFα, or IL6 production were found when pooling data for the entire groups within the cohort. Supplementary Figures S1A,B show the innate immune responses form the individual patients, where a significant difference was found. Upon PV infection of MdMs, we observed a non-significant tendency toward an increased viral burden in patients compared to controls, suggesting impaired control of PV infection in patients (Figure 4). In addition, patient MdMs showed a significantly stronger IFNβ response to PV compared to controls (Figure 3E). Supplementary Figure S2 shows the IFNβ responses in MdMs from the individual patients in whom a significant difference was measured. Finally, we correlated the IFNβ response to viral burden to demonstrate the diversity of patient IFNβ response toward PV. Interestingly, patient IFNβ responses correlated (*r* = -0.52, *p* = 0.02) with the ID50/mL, whereas controls clustered in a similar pattern, possibly reflecting elevated IFNβ responses in patient cells as a consequence of increased viral replication (Figure 3). However, this needs to be repeated in a larger cohort in order to clarify whether this picture is consistent. Importantly, we found that an individual’s age did not correlate with IFNβ responses; hence the age difference between patients and controls most probably does not have any impact on the cellular responses to infection.

To further investigate antiviral type I and III IFN responses, we infected PBMCs from eight patients and eight controls with PV (Supplementary Figure S3) and quantified IFNβ, IFNα, and IFNλ responses by Mesoscale technology. We did not observe significant differences between the individual patients and pooled controls; however, P3 and P12 demonstrated IFN responses in the upper quartile of the controls, which is in line with the RT-qPCR results in PBMCs (Supplementary Figure S1). In addition, we stimulated the PBMCs with the TLR3 agonist HMW Poly(I:C), as the TLR3 signaling pathway has been suggested to be important in the antiviral defense against PV (Oshiumi et al., 2011). Interestingly, P3 had increased IFNβ and IFNα responses compared to pooled controls (Supplementary Figures S4A,B). In contrast, P17 demonstrated a significantly impaired IFNβ response (Supplementary Figure S4A) in addition to a tendency toward decreased IFN responses following PV infection (Supplementary Figure S3). These results may suggest a possible impairment of the TLR3 pathway in P17, although no variants were identified within this pathway. However, any association between the TLR3 pathway and variants found in P17 is yet to be elucidated.

## Discussion

In the present study we used WES to identify host genetic variants in 18 patients with PPM. Variants were filtered in IVA and variant-containing genes were evaluated by function and various *in silico* tools. The major finding of this study is the identification of several rare variants involved in common antiviral processes, including innate immune responses, autophagy, and apoptosis, that we suggest might have contributed to the severe course of disease leading to PPM in these patients.

These experiments were designed based on the hypothesis that patients with PPM might have impaired immune responses toward PV. However, we did not consistently observe decreased antiviral responses; in contrast there were significantly enhanced IFNβ and CXCL10 responses in MdMs and PBMCs, respectively. The single most striking observation to emerge from our data was the correlation between IFNβ response and ID50 demonstrating that antiviral response in the patients corresponds to and likely reflects the viral burden. These data on viral replication might suggest that enhanced antiviral responses could be the consequence of increased PV replication in patient cells compared to controls. This finding broadly supports the work of other studies in this area linking type I IFN with control of primary infection, thus mapping IFN type I as a hallmark cytokine in PV infection. In addition, these results build on recent studies, including the picornavirus EV71, demonstrating the importance of type I IFN in EV71 infection (Lu et al., 2012). The observed lack of correlation in the control cohort might have several explanations. We speculate that the controls are capable of controlling the viral burden with a relative weak IFNβ response, whereas the viral replication within MdMs from a sub-group of patients resulted in high viral burden despite a similar IFNβ response. This sub-group consisted of the following patients (genes affected by variants in each patient are mentioned in brackets): P1 (*CREBBP, VPS16*), P4 (*DHX36, ULK1*), P6 (*NOS2, BNIP2*), P7 (*UBA7*, *CTSL*, and *CTSC*), P12 (*TRAF2, PTPN22, NOD1*, and *TP53BP2*), P13, and P16 (*ATG7, CHRNA5*); thus implicating variants involved in immune responses, autophagy and apoptosis as potentially disease determinants. Contrary to our expectations, we observed a high IFNβ response in Patient P5, P7 (*UBA7*, *CTSL*, *CTSC*, and *FOS*), P10 (*ULK1*, *ANXA5*, and *ARHGAP21*), P14, P15 (*CHRNG, PMAIP1*, and *VSP18*), which accounts for the significant difference between IFNβ responses to PV in MdMs between patients and controls. A possible explanation for this could be a downstream signaling defect of IFNβ or, more likely, the presence of other defects in pathways important for viral control and PV disease progression, given that the majority of identified variants belonged to common pathways involving viral restriction, apoptosis, and autophagy.

Remarkably, following PV infection in PBMCs, we did not observe a significant difference in IFNβ mRNA responses between patients and controls. These data might reflect that PBMCs are less permissive to PV than the case for MdMs despite expressing the PVR CD155 (Mendelsohn et al., 1989; Freistadt et al., 1993). In our experimental set-up, we were not able to detect any significant viral replication in the PBMCs. This supports the hypothesis that the PVR CD155 is necessary for PV infection, although not the sole determinant of tissue tropism, and productive infection (Ren and Racaniello, 1992; Ida-Hosonuma et al., 2005). Furthermore, PBMCs constitute a heterogeneous cell population with inter-individual variation; hence the small cohort size requires major differences in order to measure any significant differences. Lastly, due to redundancy in PRRs, as well as the possibility of incomplete penetrance, it might be challenging to evaluate the functional significance of variants in PBMCs. In addition, the majority of patients did not demonstrate decreased IFN responses following HMW poly(I:C) stimulation (Supplementary Figure S4). This is not necessarily surprising in light of previous studies showing normal responses to HMW poly(I:C) and viruses, even in PBMCs from autosomal dominant TLR3-deficient patients (Zhang et al., 2007). Importantly, these data do not exclude the possibility of an impaired immune response in a more biological relevant tissue, particularly when examining neuronal cells.

Noteworthy, in an *in vitro* setting, it might be difficult to answer if the phenotype is due to a defect in the peripheral innate immune response (which could be represented by PBMCs and MdMs), or if the phenotype is due to a defect in the neuronal cells targeted by the virus. We believed that by using PBMCs and MdMs, we were able to somewhat represent the peripheral innate immune response, however with the limitations described above.

An unexpected finding was the identification of 7 variants within six patients in genes involved in autophagy. The implicated genes are involved in induction and elongation of the autophagosome (*ULK1, ATG7*), lysosomal fusion (*VPS16, VPS18*), and degradation herein (*CTSL, CTSC*). Studies have found that PV can induce autophagy and utilize the components to enhance its own replication, maturation, and egress in a non-lytic manner (Jackson et al., 2005; Richards and Jackson, 2012; Bird et al., 2014). However, an antiviral effect of autophagy is in line with recent studies demonstrating targeting of PV to autophagic degradation upon cell entry (Staring et al., 2017). The direct implication of variants in the autophagy machinery in the setting of PV infection is yet to be elucidated.

It is commonly believed that the disease phenotype of PPM is a result of viral-induced cell death of the motor neurons (Girard et al., 1999; Autret et al., 2007, 2008). Remarkably, we identified 6 variants in genes involved in the apoptotic pathway. Studies have shown that PV triggers apoptosis through the mitochondrion-dependent intrinsic pathway (Belov et al., 2003), including the pro-apoptotic genes encoding TB53BP2 (variants in P2 and P12), BNIP2 (variant in P6), BNIP3 (variant in P11), and PMAIP2 (variant in P15). PV possesses several mechanisms of blocking cell death, and a variant in a pro-apoptotic gene, which would delay host cell apoptosis, could potentially act as a proviral mechanism allowing the virus more time to replicate and assemble new infectious particles. Indeed, we observed a high viral burden in MdMs from P6 and P12. These data built on studies, where blocking cell death in different cell lines upon EV-infection, including PV, all resulted in a reduction or delay in viral replication (Carthy et al., 2003; Kim et al., 2004; Autret et al., 2007, 2008).

Our findings of variants in genes encoding components of the acethylic cholinergic receptor are intriguing and of potential interest. One hypothesis is that certain defects in the acethylic cholinergic receptor and the neuromuscular endplate might predispose to severe PV infection at that location and/or confer increased vulnerability to PV, thus enhancing neuronal pathology and predisposing to tissue destruction and subsequent paralysis.

In the present study, we defined strict filtering criteria to limit the number of variants with no impact on disease progression. Notably, none of the identified variants was localized in the same gene, suggesting that predisposition to PPM may result from a number of diverse but functionally, and immunologically related signaling defects. Importantly, we found an overrepresentation of variants within genes involved in IFN signaling, autophagy, and apoptosis. Taken together, the relevance of the variants identified in WES needs further detailed functional studies in order to judge their relevance in PV infection. Noteworthily, our study only included patients surviving PV infection. The genetic profile of the patients with VDPV, or patients who died from sequelae following infection might provide further insight into PV pathogenesis. Moreover, individuals who were infected by PV during the epidemics without developing severe invasive disease in the form of PPM would have represented the optimal control population, which unfortunately was not available to us. Finally, other factors than host genetics likely influence the outcome of PV infection, including viral inoculum, virulence of the PV strain (Freistadt and Eberle, 1996; Nathanson and Kew, 2010), as well as the presence of secondary infections or immunodeficiency in the host (Abo et al., 1979; Parker et al., 2014; Aghamohammadi et al., 2017).

The final eradication of PV involves several challenges. Patients with PID were exposed to OPV excrete neurovirulent immunodeficiency-associated vaccine-derived polioviruses (iVDPVs) for months/years. These patients represent a potential reservoir for transmission of VDPV in the post-eradication era as well as being in continuous risk of PPM. Some countries have started neonatal screenings with the purpose to accelerate the diagnosis of patients with severe combined immunodeficiencies (SCIDs) and agammaglobulinemia, but these screening tests do not diagnose all rare single gene inborn errors of immunity. Furthermore, approximately 150 countries still utilize the OPV in the childhood immunization schedule, which can spread and be transmitted to PID patients incidentally. Thus, assessing the impact of rare single gene inborn errors of immunity is of critical importance to build an effective strategy for global polio eradication, including further development of potential treatments, such as antivirals exemplified by pocapavir and V-7404 (McKinlay et al., 2014; Collett et al., 2017). Importantly, research in PV immunity will not only be of relevance to the work of polio eradication. Other enteroviruses have emerged as causes of paralysis among children with major outbreaks in the Asia-Pacific region (Solomon et al., 2010). Thus, knowledge gained from studies on PV host genetics and immunity might also be valuable in the understanding of the pathogenesis underlying CNS invasion and myelitis caused by these enteroviruses.

## Data Availability

The raw data supporting the conclusions of this manuscript will be made available by the authors, without undue reservation, to any qualified researcher.

## Ethics Statement

The project was approved by the Danish National Committee in Health Research Ethics (#1-10-72-66-16) and the Danish Data protection Agency (#1-16-02-216-16) in accordance with the ethical standards of the Helsinki Declaration. Following oral and written information, all patients provided written consent prior to inclusion. The part of the project involving WES analysis of the HSE cohort, was approved by the Danish National Committee in Health Research Ethics (#1-10-72-275-15) and previously published (Mork et al., 2015).

## Author Contributions

All authors have contributed to, seen, and approved the final, submitted version of the manuscript. TM and LK conceived the idea and planned the project. N-SA collected the patient material. N-SA, SL, SJ, and MM conducted the experiments. N-SA, SL, SN, MM, and MC performed the bioinformatics analysis of the WES data. N-SA and TM drafted the first version of the manuscript.

## Conflict of Interest Statement

The authors declare that the research was conducted in the absence of any commercial or financial relationships that could be construed as a potential conflict of interest.
