# Supplementary material for: Host Genetics, Innate Immune Responses, and Cellular Death Pathways in Poliomyelitis Patients
Source: Front Microbiol. 2019 Jul 9;10:1495. doi: 10.3389/fmicb.2019.01495 (PMC6629967; doi:10.3389/fmicb.2019.01495)
Supplement: Supplementary file 1 [file Presentation_1.pdf]

Supplementary material: Host genetics, innate immune responses, and cellular death pathways in poliomyelitis patients

Authors

Nanna-Sophie B. Andersen[a,b], Simon M. Larsen[a], Sara K. Nissen[a,b], Sofie E. Jørgensen[a], Maibritt Mardahl[a], Mette Christiansen[c], Lise Kay[d], Trine H. Mogensen[a,b,e]

[a] Department of Infectious Diseases, Aarhus University Hospital, Palle Juul-Jensens Boulevard 99, 8200 Aarhus N, Denmark, [b] Department of Biomedicine, Aarhus University, Wilhelm Meyers Alle 4, 8000 Aarhus C, Denmark, [c] Department of Clinical Immunology, Aarhus University Hospital, Palle Juul-Jensens Boulevard 99, Aarhus N, 8200, Denmark, [d] Specialized hospital for Polio- and Accident Patients, Fjeldhammervej 8, 2610 Rødovre, Denmark, [e] Department of Clinical Medicine, Aarhus University Hospital, Palle Juul-Jensens Boulevard 82, 8200 Aarhus N, Denmark

Corresponding author contact information

Correspondence to Trine Hyrup Mogensen, Department of Infectious Diseases, Aarhus University Hospital, Palle Juul-Jensens Boulevard 99, 8200 Aarhus N, Denmark. Tel: +45 20125280; e-mail: trinmoge@rm.dk

Supplementary Methods 1: Detailed description of the filtering process

Filter Description starting with 403,827 variants spanning 20,789 genes.

The filter settings can be exported upon request.

Confidence filter

Variants were kept with call quality at least 30.0 AND with read depth at least 25.0 AND with allele fraction at least 35.0 with genotype quality at least 30.0 AND outside top 5.0% most exonically variable 100 base windows in healthy public genomes (1000 genomes) AND outside top 1.0% most exonically variable genes in healthy public genomes (1000 genomes).

Explanation of "confidence filter"

Call quality at least 30.0, read depth at least 25.0, allele fraction at least 35.0, and genotype quality at least 30.0 are modified from default settings in the software. These values provide one of the highest possible qualities. A high call quality ensures that variants are placed correctly in the genome. A high read depth ensures that the sequence are aligned at least 25 times, hereby excluding random sequencing errors. We included a filter providing information of the allele fraction. In theory, if a patient is homozygous, the fraction will be 100%, and if the patient is heterozygous, the fraction will be 50%. In reality, this filter is applied with a lower cut-off fraction as the sequencing of each allele can vary depending on the efficiency of amplification

Variants were excluded if they were outside top 5.0% most exonically variable 100 base windows in healthy public genomes (1000 genomes) AND outside top 1.0% most exonically variable genes in healthy public genomes (1000 genomes). This filter excludes known mutational hotspots.

Common variants

Variants were excluded that are observed with an allele frequency greater than or equal to 0.1% of the genomes in the 1000 genomes project, the NHLBI ESP exomes (All), the ExAC Frequency, or the gnomAD Frequency.

Explantion of the filter "common variants"

We searched for rare variants contribution to PPM phenotype. The frequency of PPM is 0.1 – 1%, hence the cut-off should be below this frequency.

Predicted deleteriousness filter I

Variants were kept (up to 2 bases into intron) that are experimentally observed to be associated with a phenotype: Pathogenic, Possibly Pathogenic OR Disease-associated according to HGMD OR established gain of function in the literature OR Frameshift, in-frame indel, or stop codon change OR Missense unless predicted to be innocuous by SIFT or PolyPhen-2 OR predicted deleterious by having CADD score > 15.0 OR predicted to disrupt splicing by MaxEntScan OR in promoter binding site.

Explanation of "Predicted deleteriousness filter I"

The terms "Pathogenic" or "Possibly pathogenic" are used by The American College of Medical Genetics and Genomics (ACMG) [1]. Variants were kept if they were disease associated according to computed ACMG guidelines classification. The guidelines recommends the use of specific standard terminology "pathogenic", "likely pathogenic", "uncertain significance", "likely benign", and "benign" to describe variants identified in genes that cause Mendelian disorders.

The PolyPhen-2 and SIFT scores are explained elsewhere.

Genetic analysis

Variant were excluded if they occurred in 5 of the case samples

Explanation: In our study, we searched for rare variants to eliminate false positive variants that are common among the general population. When multiple identical variants with very low or abscent frequency in the GnomAD database were found, these were manually curated, and these turned out not to be genuine variants, but artefacts of the sequencing procedure, generally present in repetitive regions. For this reason, we excluded variants if they occurred in more than 5 case samples, but we did not exclude non-identical variants that were present in the same gene or signalling pathway.

Biological filter

See separate **Table S2**

Predicted deleteriousness filter II

Variants were kept (up to 2 bases into intron) that are Frameshift, in-frame indel, or stop codon change OR predicted deleterious by having CADD score > 15.0 OR predicted to disrupt splicing by MaxEntScan

Predicted deleteriousness filter III

Variant were kept (up to 2 bases into intron) that are predicted to disrupt splicing by MaxEntScan OR in evolutionary-conserved region with a phyloP p-value of less than or equal to 0.01.

Explanation of the filters “Predicted deleteriousness filter”

The deleterious filter I, II and III somewhat overlap. This is deliberately and important because the software circumvent the filters if the filter section contains “OR”. By adding the filter in an additional and separate section, we are able to generate a filtration complying with well established parameters. In summary; Variants were kept (up to 2 bases into intron) that are experimentally observed to be associated with a phenotype: Pathogenic, Possibly Pathogenic OR Disease-associated according to HGMD OR established gain of function in the literature OR Frameshift, in-frame indel, or stop codon change OR Missense unless predicted to be innocuous by SIFT or PolyPhen-2 **IF** predicted deleterious by having CADD score > 15.0 **OR IF** predicted to disrupt splicing by MaxEntScan OR in promoter binding site.
The phyloP p-value is default, and adjusting this value to a lower number, did not make a relevant difference.

Software version used:
Ingenuity Variant Analysis version 5.2.20180419
Content versions: CADD (v1.3), CentoMD (4.1), EVS (ESP6500SI-V2), Allele Frequency Community (2018-01-17), JASPAR (2013-11), Ingenuity Knowledge Base Snapshot Timestamp (2018-04-19 20:56:59.0), Ingenuity Knowledge Base (Pandora 180419.002), Vista Enhancer (2012-07), OMIM (May 26, 2017), gnomAD (2.0.1), Clinical Trials (Pandora 180419.002), BSIFT (2016-02-23), TCGA (2013-09-05), PolyPhen-2 (v2.2.2), 1000 Genome Frequency (phase3v5b), Clinvar (2018-01-03), DGV (2016-05-15), COSMIC (v83), ExAC (0.3.1), HGMD (2017.4), PhyloP (2009-11), DbSNP (150(2017-07-10)), TargetScan (6.2), SIFT4G (2016-02-23).

Supplementary Method 2: Detailed description of the variant containing genes

Variants-affected genes are here described in details regarding their function. Genes with similar functions are categorized together. A few variants are labelled with more than one category (Table 1), but will only be described once.

1. Variant-affected genes within intracellular immune signalling

Several genes encoding molecules with potential impact on innate sensing pathways and downstream type I IFN and pro-inflammatory cytokine production were identified (Table 1). NOD1, a pattern recognition receptor (PRR) that normally senses bacterial peptidoglycans, has also been described to recognize dsRNA from HCV as well as being involved in the inflammatory response to dsRNA [2]. These results demonstrate the complexity of the cross talk between different classes of PRRs and may have relevance in PV infection. Furthermore, DHX36 has also been described to sense dsRNA, though in a complex with DDX1, DDX2 and TRIF leading to type I IFN response following influenza A or reovirus infection [3]. Additionally, CC2D1A/TAPE regulates the TLR3 pathway, thus potentially playing an important role in regulation of antiviral responses [4]. Furthermore, Tumor necrosis factor receptor-associated factor (TRAF) 3 and 6 act as scaffolding proteins by recruiting downstream kinases TBK1 or IKKα/β/γ complex, eventually leading to IRF3 or NF-κB activation. Induction of USP25 by RNA or DNA virus infection promotes antiviral responses by mediating the stabilization of TRAF3 and TRAF6 [5]. In addition, PTPN22 binds TRAF3 hereby regulating type I IFN and proinflammatory responses [6]. TRIM67 is less well-described, but TRIMs are known to increase innate immune responses in HEK293T cells, and interestingly TRIM67 is mainly expressed in neuronal tissue [7]. TNIP1 and TRAF2 have been described with immune modulatory effects by regulation NF-κB [8, 9], and have also been shown to participate in regulation of viral replication within the host cell [10, 11]. Furthermore, TRAF2 functions as a mediator of the anti-apoptotic signals

from TNF receptors [12]. Lastly, CREBBP increases expression of IFNβ promoter [13], and FOS is involved in AP-I transcription.

2. Variant-affected genes regulating viral infectivity and replication

We identified eight variants that potentially could impact viral replication in cells of the immune system as well as neuronal cells (Table 1). Little is known of PV restriction factors in humans; however, MX1 [14] has mainly been described as an Interferon-stimulated gene (ISG) in mice. Moreover, NOS2 that have been suggested to inhibit replication of mouse cytomegalovirus [15]. Furthermore, ISG15 including the cognate ISG15 E1 activating enzyme, UBA7, has been shown to decrease replication of Influenza A and HCV [16]. ANXA5 and ANXA6 inhibits HIV and Influenza A replication respectively [17, 18], which both are RNA viruses like PV. Additionally, GBP1 have been shown to decrease viral replication of several RNA viruses [19, 20]. Lastly, ARHGAP21 plays a role in regulating the neuraminidase A (NA) transport of Influenza A, thereby impacting on viral assembly and replication [21]

3. Variant-affected genes in the autophagy pathway

An interesting finding was the identification of seven variants within six individual patients in genes involved in autophagy (Table 1). Initiation of macro-autophagy (referred to as autophagy) is achieved by the activation of the ULK1-complex, which subsequently activates the Beclin 1-VPS34 complex leading to the generation of a double-membraned phagophore. Elongation and completion of the phagophore into the complete autophagosome depends on two ubiquitin-like (UBL) conjugation reactions, both involving ATG7, which is a homolog of the E1 enzyme. First, ATG12 is activated by ATG7 and subsequently conjugated to ATG5 by ATG10. Secondly, ATG7 takes part in the LC3 lipidation, which is required for recruitment of membranes used in the closure of the autophagosome.

Next, the autophagosome is targeted for fusion with the lysosome and degradation of its contents. VPS16 and VPS18 are both part of the HOPS complex, which is recruited to the autophagosomal membrane, where it interacts with syntaxin-17 and fuses with the lysosome [22, 23]. Within the lysosome, various proteases execute the degradative function of this organelle. Cathepsin-L (CTSL) and Cathepsin-C (CTSC) are both cysteine proteases found within the lysosome [24], and studies have shown them being relevant in autophagy [25, 26]

4. Variant-affected genes influencing apoptosis through the mitochondrion-dependent intrinsic pathway

Enteroviruses are known both to induce and delay host cell death, and their control of the cell survival/death-balance is crucial for viral survival and replication in human cell [27]. Interestingly, we identified five variants in genes involved in the mitochondrion-dependent intrinsic apoptotic pathway, including BNIP2, BNIP3, PMAIP1, and two different variants in the gene encoding TBP53BP2 (Table 1). BNIP2, BNIP3, and PMAIP1 belongs to a family of pro-apoptotic proteins in the BCL-2 family [28]. Studies have demonstrated a significant reduction in coxackievirus replication due to the pro-apoptotic effect of BNIP2 in host cells [29]. PMAIP1 has been demonstrated to be an important component of the innate immune response to viral infection, leading to enhanced cellular apoptosis following infection [30]. Indeed, variants in pro-apoptotic genes may compromise the host cell ability to limit viral dissemination [31].

5. Variant-affected genes in the nicotinerg acethylcholine receptor complex

Common for five patients in the cohort were the finding of variants encoding subunits of the nicotinic acetylcholine receptor (nAChR) (Table 1). The nAChR is composed by five transmembrane subunits, which confer different physiological properties throughout the body [32, 33]. CHRNA1 encodes the

α1 subunit, which is part of the nAChR found in the neuromuscular junction. Furthermore, it harbours the antigenic site involved in the pathogenesis of Myasthenia Gravis. CHRNG encodes the γ-subunit found in the fetal nAChR prior to innervation of the muscle. Re-expression of this subunit has been shown in denervated or paralyzed muscle. The α5 subunit (CHRNA5) is found in the brain, especially dopaminergic neurons of the ventral tegmental area (VTA), and has been associated to nicotine dependence [34]. The α7 subunit (CHRNA7) is expressed in various neurons but also non-neuronal cells, including lymphocytes, macrophages and dendritic cells, and it has been suggested to play a role in regulation of the immune system and CNS inflammation [35-37]. The α10 subunit (CHRNA10) has mostly been described in hair cells in the olivocochlear system of the inner ear, but they have also been found in human lymphocytes [38, 39].

6. Variant-affected genes with no uniform classification

We identified four variants in the four genes encoding MMP2, MMP8, C3AR1 and ERAP1 (Table 1). The Matrix metalloproteinases MMP2 and MMP8 have been described important in various biological settings, including the blood brain barrier integrity and cell signalling [40]. The gene *C3AR1* encodes the C3a anaphylatoxin chemotactic receptor involved in the alternative pathway of the complement system, a part of the innate immunesystem [41]. In addition, recent studies indicate that C3a through interaction with C3aR1 may have a role in intestinal regeneration by enhancement of stem cell expansion and organoid formation [42]. Breakdown of the mucosal integrity will allow pathogens, such as PV, to enter the blood stream indiscriminately. Lastly, a variant in ERAP1, an aminopeptidase that trims peptides for the MHC class I antigen pathway, could impair the host cell ability to present viral peptides to the cytotoxix T lymphocytes [43].

Table S1: Patient characteristics

Patient ID	Age upon inclusion (years)	Age upon infection	Gender	Clinical PPM phenotype
**1**	58	EC	Male	One leg
**2**	66	8 months	Female	Whole body
**3**	69	EC	Male	Whole body, respiration
**4**	75	1 year	Male	Both legs
**5**	81	5 years	Female	One leg
**6**	75	8 months	Female	Both legs
**7**	77	3 years	Female	One leg
**8**	71	EC	Female	One leg
**9**	68	EC	Male	Whole body
**10**	66	EC	Male	One arm, one leg
**11**	76	EC	Male	Whole Body
**12**	80	3.5 years	Male	Both legs
**13**	71	2 years	Male	Whole body
**14**	68	2.5 years	Male	Lower body, both legs
**15**	71	1.5 years	Male	Back, respiration
**16**	81	12 years	Female	Whole body
**17**	68	N/A	Male	Both legs
**18**	49	N/A	Female	One leg

Table S1. The cohort consisted of 18 patients (seven females, eleven males), with a mean age of 71 years at time of inclusion. None of the patients fulfilled the criteria of post-polio syndrome which include a period of recovery. We believe non-post polio patients potentially suffered from a more severe infection compared to patients who had years of recovery.

P1-P17 were of Caucasian origin, whereas P18 was of Asian origin. Disease histories were obtained by patient memory since medical files from the time of infection were not available. Briefly, P6, acquired a nosocomial infection; P11 was infected while having another childhood disease; P14 was infected at the hospital following a trauma; P13 were infected at a birthday party where nine of sixteen children were infected of whom 2 died; and, P16 reported having severe infections of all childhood diseases. *PPM*: paralytic poliomyelitis; *whole body*: includes legs, arms, back, and abdomen. Only P15 and P3 reported difficulties in the respiratory muscles; *EC*: early childhood; *N/A:* patients did not remember.

Table S2: Genes included in the biological filter

ABCA1	ABCB1	ABCB10	ABCB11	ABCB4	ABCB5	ABCC1	ABCC2	ABCC3	ABCC4
ABCC6	ABCC8	ABCC9	ABCG1	ABCG2	ABCG5	ABHD3	ABHD6	ABL1	ABLIM3
ACADVL	ACKR2	ACKR3	ACKR4	ACOT1	ACP2	ACP4	ACP5	ACP6	ACPP
ACTA1	ACTA2	ACTB	ACTC1	ACTG1	ACTG2	ACTL6B	ACVR1	ACVR1B	ACVR1C
ACVR2A	ACVR2B	ACVRL1	ADA	ADA2	ADAM10	ADAM15	ADAM17	ADAR	ADCYAP1
ADIPOQ	ADORA2A	ADORA2B	ADORA3	ADRA1A	ADRA1B	ADRA1D	ADRA2A	ADRA2B	ADRA2C
ADRB1	ADRB2	AGBL4	AGBL5	AGER	AGFG1	AGO1	AGO2	AGO3	AGO4
AGRN	AGTRAP	AHCYL1	AHNAK	AHR	AICDA	AIM2	AIP	AKAP13	AKT1
AKT2	AKT3	AKTIP	ALB	ALCAM	ALDH1L1	ALOX5	ALPI	ALPL	ALPP
ALPPL2	ALS2	AMACR	AMPD1	AMPH	ANGPT1	ANKRD17	ANPEP	ANXA1	ANXA5
ANXA6	AP1M1	AP1S1	AP2M1	APBB1IP	APC2	APCS	APEX1	APH1A	APH1B
APOA1	APOA2	APOA4	APOB	APOBEC1	APOBEC3A	APOBEC3B	APOBEC3C	APOBEC3D	APOBEC3F
APOBEC3G	APOBEC3H	APOC1	APOC2	APOC3	APOC4	APOD	APOE	APOF	APOL1
APOM	APP	AR	ARAF	ARCN1	AREG	ARG1	ARHGAP21	ARHGAP33	ARHGDIB
ARID1A	ARID5A	ARL16	ARNTL	ARRB1	ARTN	ASF1A	ATCAY	ATF2	ATF4
ATG10	ATG12	ATG13	ATG16L1	Atg2b	ATG3	ATG4A	ATG4B	ATG4C	ATG4D
Atg5	ATG7	ATG9A	ATG9B	ATM	ATP1A2	ATP1B3	ATP2C1	ATP5B	ATP6AP1
ATP6AP2	ATP6V0A2	ATP6V0B	ATP6V0C	ATP6V0D1	ATP6V1A	ATP6V1B2	ATP6V1G1	ATR	ATRX
ATXN1	AXL	AZI2	AZU1	B2M	BACH2	BAG6	BAIAP3	BAK1	BANF1
BANK1	BARHL2	BATF	BATF3	BAX	BCL10	BCL11B	BCL2	BCL2L1	BCL2L11
BCL3	BCL6	BCLAF1	BCR	BDNF	BECN1	BHLHE40	BHMT	BICD1	BIRC2
BIRC3	BLK	BLNK	BLOC1S3	BLOC1S6	BMP2	BMP2K	BMP4	BMP7	BMP8A
BMPR2	BMX	BNIP2	BNIP3	BNIP3L	BPIFA1	BRCA1	BRCA2	BRINP1	BSG
BST2	BTF3	BTK	BTLA	BTN1A1	C10orf99	C19orf57	C19orf66	C1orf106	C1QA
C1QB	C1QC	C1QTNF7	C1RL	C1S	C2	C3	C3AR1	C4A/C4B	C4BPA
C4BPB	C5	C5AR1	C5AR2	C6	C6orf15	C7	C8A	C8B	C9
CACNA1A	CACNA1C	CACNA1D	CACNA1E	CACNA1F	CACNA1S	CACNA2D1	CACNA2D2	CACNA2D3	CACNA2D4
CACNB1	CACNB2	CACNB3	CACNB4	CACNG1	CACNG2	CACNG3	CACNG4	CACNG5	CACNG6
CACNG7	CACNG8	CAD	CADM1	CALCOCO2	CALR	CAMK2B	CAMK2D	CAMK4	CAMP
CANT1	CAPN3	CARD11	CARD9	CASP1	CASP10	CASP12	CASP14	CASP2	CASP3
CASP4	CASP5	CASP6	CASP7	CASP8	CASP9	CASQ1	CAT	CAV1	CBLB
CBLL1	CBS/CBSL	CC2D1A	CCDC130	CCDC88B	CCL1	CCL11	CCL13	CCL14	CCL15
CCL16	CCL17	CCL18	CCL19	CCL2	CCL20	CCL21	CCL22	CCL23	CCL24
CCL25	CCL26	CCL27	CCL28	CCL3	CCL3L1	CCL3L3	CCL4	CCL5	CCL7
CCL8	CCNA1	CCNA2	CCNB3	CCND1	CCNK	CCNT1	CCR1	CCR10	CCR2
CCR3	CCR4	CCR5	CCR6	CCR7	CCR8	CCR9	CCRL2	CCT5	CD163
CD180	CD19	CD1A	CD1B	CD1C	CD1D	CD1E	CD2	CD200	CD207
CD209	CD22	CD226	CD24	CD244	CD247	CD27	CD274	CD276	CD28
CD300LF	CD36	CD38	CD3D	CD3E	CD3G	CD4	CD40	CD40LG	CD44
CD46	CD47	CD48	CD5	CD52	CD55	CD58	CD59	CD6	CD69
CD7	CD70	CD72	CD74	CD79A	CD79B	CD80	CD81	CD83	CD86

CD8A	CD8B	CD93	CD99	CDC42	CDC42BPA	CDC42BPB	CDC73	CDK2	CDK4
CDK5R1	CDK6	CDKN1A	CDKN1B	CDKN2A	CEACAM1	CEACAM5	CEACAM6	CEBPB	CEL
CEP68	CER1	CES1	CFB	CFD	CFH	CFHR1	CFHR5	CFI	CFL1
CFL2	CFLAR	CGAS	CH25H	CHD1	CHMP2A	CHMP3	CHMP4B	CHMP4C	CHP1
CHRM1	CHRM2	CHRM3	CHRM4	CHRM5	CHRNA1	CHRNA10	CHRNA2	CHRNA3	CHRNA4
CHRNA5	CHRNA6	CHRNA7	CHRNA9	CHRNB1	CHRNB2	CHRNB3	CHRNB4	CHRND	CHRNE
CHRNG	CHST5	CHUK	CIITA	CISH	CIT	CKB	CKLF	CKM	CKMT2
CLC	CLCF1	CLDN3	CLEC1B	CLEC2D	CLEC4D	CLEC4E	CLEC4G	CLEC4M	CLEC5A
CLEC6A	CLEC7A	CLIC4	CLK1	CLSTN2	CLSTN3	CLTCL1	CLU	CLUH	CMTM2
CMTM3	CMTM4	CMTM5	CMTM6	CMTM7	CMTM8	CNNM1	CNOT7	CNR1	CNR2
CNTF	COCH	COL10A1	COL11A2	COL17A1	COL18A1	COL1A1	COL1A2	COL28A1	COL2A1
COL3A1	COL5A3	COLEC10	COLEC12	COPA	COPB1	COPB2	COPG1	COPS5	CORO1A
CORT	COX6A1	CPSF4	CPT2	CR1	CR1L	CR2	CRAMP1	CRBN	CREB1
CREB3	CREB3L3	CREBBP	CREBZF	CRH	CRP	CRTAM	CRTC2	CRY1	CRY2
CRYAB	CS	CSE1L	CSF1	CSF2	CSF2RB	CSF3	CSF3R	CSH1/CSH2	CSHL1
CSK	CSMD1	CST3	CTBP1	CTBP2	CTF1	CTLA4	CTNNB1	CTSA	CTSB
CTSC	CTSD	CTSE	CTSF	CTSG	CTSH	CTSK	CTSL	CTSO	CTSS
CTSV	CTSW	CTSZ	CX3CL1	CX3CR1	CXADR	CXCL1	CXCL10	Cxcl11	CXCL12
CXCL13	CXCL14	CXCL16	CXCL17	CXCL2	CXCL3	CXCL5	CXCL6	CXCL8	Cxcl9
CXCR1	CXCR2	CXCR3	CXCR4	CXCR5	CXCR6	CYBA	CYBB	CYLD	CYP17A1
CYP1A1	CYP1B1	CYP2D6	CYP2U1	CYP3A4	CYP51A1	CYTL1	DAG1	DAPK2	DAPP1
DAXX	DBT	DCK	DCLK1	DCLK2	DCT	DDB1	DDIT3	DDIT4	DDOST
DDX1	DDX21	DDX3X	DDX41	DDX5	DDX58	DDX6	DDX60	DEF6	DEFA4
DEFA5	DEFB114	DEFB118	DEFB127	DEK	DGCR8	DGKB	DHFR	DHODH	DHRS2
DHX36	DHX38	DHX58	DHX9	DIABLO	DICER1	DIRAS3	DKK1	DKK3	DLG1
DLG2	DLL1	DLL4	DMAP1	DMBT1	DNAH10	DNAJA1	DNAJB1	DNAJB11	DNAJB12
DNAJB13	DNAJB14	DNAJB2	DNAJB3	DNAJB4	DNAJB5	DNAJB6	DNAJB7	DNAJB8	DNAJB9
DNAJC1	DNAJC10	DNAJC11	DNAJC12	DNAJC13	DNAJC14	DNAJC15	DNAJC16	DNAJC17	DNAJC18
DNAJC19	DNAJC2	DNAJC21	DNAJC22	DNAJC24	DNAJC25	DNAJC27	DNAJC28	DNAJC3	DNAJC30
DNAJC4	DNAJC5	DNAJC5B	DNAJC5G	DNAJC6	DNAJC7	DNAJC8	DNAJC9	DNASE1	DNM1L
DOCK2	DOK3	DPP4	DRD3	DRD5	DROSHA	DTX1	DTX2	DTX3	DTX3L
DUOX2	DUSP1	DUSP10	DUSP14	DUSP22	DUSP27	DUSP3	DYRK1A	E2F2	EBF1
EBI3	ECM1	ECSIT	EDA	EDN1	EED	EEF1A1	EEF1G	EFNB2	EGFR
EGR1	EGR2	EGR3	EHMT2	EIF2AK2	EIF2AK3	EIF2AK4	EIF2S1	EIF3A	EIF3C
EIF3G	EIF3L	EIF4A3	EIF4EBP1	EIF4EBP2	ELANE	ELAVL1	ELF1	ELK1	ELMO1
ELP1	ENG	ENGASE	ENO1	ENTPD7	EP300	EPAS1	EPHA4	EPHB2	EPHB6
EPO	EPS8L3	ERAP1	ERBB2	ERCC1	ERCC2	ERCC5	EREG	ERN1	ESR1
ESR2	ETS1	EXOC2	EXOSC4	EXOSC5	EZH2	F11R	F12	F13A1	F2
F2RL1	F5	F8	FADD	FAM111A	FAM3B	FAM3C	FAM3D	FAM49B	FANCG
FARP1	FAS	FASLG	FASN	FAU	FBN1	FBRS	FBXO44	FBXO9	FBXW10
FCAMR	FCAR	FCER1A	FCER1G	FCER2	FCGR1A	FCGR1B	FCGR2A	FCGR2B	FCGR2C
FCHO2	FCN1	FCN2	FCN3	FCRL3	FDPS	FERMT3	FFAR2	FFAR3	FFAR4
FGA	FGB	FGF1	FGF2	FGF4	FGF7	FGFR1	FGFR2	FGFR3	FGFR4

FGG	FGR	FKBP1A	FLG	FLT3	FLT3LG	FN1	FNBP1	FOLR1	FOS
FOSL1	FOXA1	FOXA2	FOXA3	FOXD1	FOXJ1	FOXO1	FOXO3	FOXP3	FPGS
FPR1	FPR2	FRS2	FSCN1	FSHB	FUBP1	FURIN	FUS	FXR1	FYN
G3BP1	G6PD	GAB1	GABARAPL1	GABRA1	GABRA3	GABRA4	GABRA5	GABRA6	GABRB1
GABRB2	GABRB3	GABRE	GABRG1	GABRG2	GABRG3	GABRP	GABRR3	GAD1	GAD2
GADD45A	GADD45B	GAK	GALC	GAS6	GATA3	GBP1	GBP2	GBP5	GCLC
GFAP	GFI1	GFI1B	GGH	GH1	GH2	GHRH	GHRHR	GIMAP1	GIMAP2
GIMAP4	GIPR	GJA1	GJA4	GLB1	GLG1	GLYR1	GNRH1	GNRH2	GNRHR
GOLGA4	GOPC	GOT1	GOT1L1	GOT2	GP2	GPAM	GPNMB	GPR146	GPR174
GPT	GPT2	GPX1	GPX4	GRB2	GRHPR	GRIA1	GRIA2	GRIA3	GRIA4
GRIN2C	GRK2	GRK5	GRK6	GRM4	GRN	GSK3B	GSTA1	GTF2F1	GUCY1A2
GUCY1A3	GUCY1B3	GUCY2C	GUCY2D	GUCY2F	GZMA	HARBI1	HAVCR1	HAVCR2	HBZ
HCFC2	HCK	HCST	HDAC1	HDAC10	HDAC11	HDAC2	HDAC3	HDAC4	HDAC5
HDAC6	HDAC7	HDAC8	HDAC9	HDC	HECTD1	HELB	HERC5	HEXB	HGF
HIF1A	HIPK1	HIPK3	HIRA	HIST1H1B	HIST1H1C	HIST1H1D	HIST1H1E	HIST1H2BN	HIST1H2BO
HIST1H4A	HIST1H4B	HIST1H4C	HIST1H4D	HIST1H4F	HIST1H4H	HIST1H4I	HIST1H4J	HIST1H4K	HIST1H4L
HIST2H4A	HIST2H4B	HIST4H4	HLA-A	HLA-B	HLA-C	HLA-DMA	HLA-DMB	HLA-DOA	HLA-DOB
HLA-DPA1	HLA-DPB1	HLA-DQA1	HLA-DQA2	HLA-DQB1	HLA-DQB2	HLA-DRA	HLA-DRB1	HLA-DRB3	HLA-DRB4
HLA-DRB5	HLA-E	HLA-F	HLA-G	HMGA1	HMGB1	HMGB2	HMGCR	HMGN2	HMOX1
HNF4A	HNRNPM	HOXA7	HPGD	HPRT1	HPS1	HPX	HRAS	HRH1	HRH2
HRH3	HRH4	HSCB	HSF1	HSF4	HSH2D	HSP90AA1	HSP90AB1	HSP90B1	HSPA12A
HSPA12B	HSPA13	HSPA14	HSPA1L	HSPA2	HSPA4	HSPA4L	HSPA5	HSPA6	HSPA8
HSPA9	HSPB1	HSPB11	HSPB2	HSPB3	HSPB6	HSPB7	HSPB8	HSPB9	HSPD1
HSPE1	HSPH1	HTATSF1	HTR4	HYAL1	HYAL2	HYAL3	HYAL4	HYOU1	ICAM1
ICAM3	ICOS	ID1	ID2	IDO1	IFI16	IFI27	IFI30	IFI44	IFI44L
IFI6	IFIH1	IFIT1	IFIT1B	IFIT2	IFIT3	IFIT5	IFITM1	IFITM2	IFITM3
IFNA10	IFNA14	IFNA16	IFNA17	IFNA2	IFNA21	IFNA4	IFNA5	IFNA6	IFNA7
IFNA8	IFNAR1	IFNAR2	IFNB1	IFNE	IFNG	IFNGR1	IFNGR2	IFNK	IFNL1
IFNL2	IFNL3	IFNL4	IFNLR1	IFNW1	IGF1	IGF1R	IGF2R	IK	IKBKB
IKBKE	IKBKG	IKZF4	IL10	IL10RA	IL10RB	IL11	IL12A	IL12B	IL12RB1
IL12RB2	IL13	IL15	IL15RA	IL16	IL17A	IL17B	IL17C	IL17D	IL17F
IL17RA	IL17RC	IL18	IL18BP	IL18R1	IL18RAP	IL19	IL1A	IL1B	IL1F10
IL1R1	IL1R2	IL1RAP	IL1RAPL1	IL1RAPL2	IL1RL1	IL1RL2	IL1RN	IL2	IL20
IL20RB	IL21	IL21R	IL22	IL23A	IL23R	IL24	IL25	IL26	IL27
IL27RA	IL2RA	IL2RB	IL2RG	IL3	IL32	IL33	IL36A	IL36B	IL36G
IL36RN	IL37	IL4	IL4R	IL5	IL6	IL6R	IL6ST	IL7	IL7R
IL9	IL9R	ILF3	IMPDH1	IMPDH2	INHBA	INPP5D	INPPL1	INS	INSL3
IPO7	IRAK1	IRAK2	IRAK3	IRAK4	IRF1	IRF2	IRF2BP1	IRF3	IRF4
IRF5	IRF6	IRF7	IRF8	IRF9	IRGM	IRS1	IRS2	ISG15	ISG20
ITCH	ITGA2	ITGA3	ITGA4	ITGA5	ITGA9	ITGAD	ITGAL	ITGAM	ITGAV
ITGAX	ITGB1	ITGB2	ITGB3	ITGB5	ITGB8	ITK	ITLN1	ITM2B	ITPKB
ITPKC	IVNS1ABP	JAG1	JAK1	JAK2	JAK3	JCHAIN	JUN	KANSL1	KATNB1
KCNA3	KCNA4	KCNA5	KCNA7	KCNE1	KCNE2	KCNG2	KCNH2	KCNIP4	KCNJ12

KCNJ2	KCNJ3	KCNJ5	KCNJ8	KCNK2	KCNK3	KCNN3	KCNN4	KCNRG	KIDINS220
KIF11	KIF1A	KIF2A	KIF5A	KIR2DL2	KIR2DL4	KIR2DL5A	KIR2DL5B	KIR2DS3	KIR3DL1
KIR3DL2	KIR3DL3	KIR3DS1	KISS1	KISS1R	KIT	KITLG	KL	KLB	KLF2
KLF4	KLK3	KLK9	KLRB1	KLRC1	KLRC2	KLRD1	KLRF1	KMT2D	KPNB1
KRT16	KRT20	KRT8	KRTAP9-9	KSR1	KSR2	L2HGDH	LAG3	LAMP1	LAMP2
LAMTOR5	LANCL1	LAPTM5	LARP1	LAT	LAT2	LAX1	LBP	LBR	LCAT
LCK	LCN2	LCP2	LDHA	LDHB	LDHC	LDLR	LEP	LGALS1	LGALS3
LGALS9	LGALS9B	LGI1	LHB	LHX3	LIF	LIFR	LIG4	LILRB1	LILRB2
LILRB3	LIMK1	LIMK2	LINGO1	LNX1	LONP1	LPA	LRP1B	LRRC23	LRRK2
LSM1	LSM14A	LSR	LTA	LTB	LTBR	LTF	LY6E	LY96	LYN
LYST	LYZ	MAFK	MAGEE2	MAGT1	MALAT1	MALT1	MAML1	MAN2B1	MAP1LC3A
MAP1LC3B	MAP1LC3B2	MAP1LC3C	MAP2K1	MAP2K2	MAP2K3	MAP2K4	MAP2K5	MAP2K6	MAP2K7
MAP3K1	MAP3K12	MAP3K14	MAP3K7	MAP3K7CL	MAP3K8	MAP4K1	MAP4K4	MAPK1	MAPK10
MAPK11	MAPK12	MAPK13	MAPK14	MAPK15	MAPK3	MAPK4	MAPK6	MAPK7	MAPK8
MAPK9	MAPKAPK2	MAPT	MARCH2	MARCO	MARK1	MARK2	MASP2	MATN3	MAVS
MBD2	MBL2	MBP	MC2R	MCL1	MDK	MDM2	MED11	MED14	MED21
MED26	MED27	MED28	MED30	MED6	MED7	MEF2C	MEFV	MERTK	MFN1
MGAT1	MGAT5	MGAT5B	MGLL	MGMT	MGST1	MICA	MICB	MID1	MID1IP1
MID2	MIF	MINK1	MIR17HG	MITD1	MKNK1	MKRN3	MLANA	MLH1	MLST8
MME	MMP12	MMP19	MMP2	MMP3	MMP8	MMP9	MNAT1	MNDA	MNS1
MNX1	MOG	MOK	MORN1	MOV10	MPO	MPZ	MR1	MS4A1	MSH2
MSR1	MSRB1	MST1R	MTDH	MTHFD2	MTHFR	MTHFS	MTOR	MTR	MTRR
MUC5B	MUS81	MVK	MVP	MX1	MX2	MYC	MYD88	MYDGF	MYLK
MYO5B	MYOD1	NAA10	NAALADL2	NAMPT	NBN	NBR1	NCBP3	NCF1	NCF1C
NCF2	NCF4	NCK1	NCL	NCOA2	NCOA3	NCOR1	NCOR2	NCR1	NCR2
NCR3	NCR3LG1	NCSTN	NDFIP1	NDP	NECTIN4	NEIL1	NEK8	NEK9	NFAT5
NFATC1	NFATC2	NFATC3	NFATC4	NFE2L2	NFIL3	NFKB1	NFKB2	NFKBIA	NFKBIB
NFKBID	NFKBIE	NFKBIZ	NGF	NGFR	NINJ1	NLRC3	NLRC4	NLRC5	NLRP10
NLRP12	NLRP14	NLRP2	NLRP3	NLRP4	NLRP5	NLRP6	NLRP9	NLRX1	NMI
NOD1	NOD2	NOP53	NOS2	NOTCH1	NOTCH2	NOTCH3	NOTCH4	NOX1	NOX3
NOX4	NPC2	NPM1	NPY	NPY1R	NR2F1	NR3C1	NR4A1	NR4A2	NR4A3
NRAS	NRBP1	NRF1	NRG1	NT5C3A	NT5E	NTF3	NTF4	NTHL1	NTRK2
NUDCD3	NUP153	NUP205	NUP214	NUP54	NUP62	NUP98	NXF1	OAS1	OAS2
OAS3	OASL	ODC1	ODF1	OGG1	OMA1	OPN1SW	OPRK1	OPRM1	OPTN
ORAI1	ORM1	ORM2	OSM	OTUB1	OTUB2	OTUD5	OTUD7B	OTULIN	OXSR1
P2RX1	P2RX2	P2RX3	P2RX4	P2RX5	P2RX6	P2RX7	P2RY1	P2RY2	P2RY6
PAEP	PAFAH1B1	PAFAH1B2	PAFAH1B3	PAG1	PAK1	PAK2	PAK3	PANX1	PARD6A
PARP1	PARP10	PARP11	PARP12	PARP9	PAX5	PBK	PBX1	PCBP2	PCDH18
PCSK7	PCYOX1	PDCD1	PDCD1LG2	PDCD4	PDCD6IP	PDE4A	PDE4B	PDE4C	PDE4D
PDE8A	PDGFC	PDGFRL	PDZD2	PEBP4	PECAM1	PELI1	PF4	PF4V1	PFDN1
PFDN6	PGAP3	PGF	PGLYRP2	PHB2	PHF2	PI4KA	PI4KB	PIBF1	PIK3C2A
PIK3C2B	PIK3C2G	PIK3C3	PIK3CA	PIK3CB	PIK3CD	PIK3CG	PIK3R1	PIK3R2	PIK3R3
PIK3R4	PIK3R5	PIK3R6	PIKFYVE	PIM2	PIN1	PINK1	PIP5K1B	PIP5K1C	PKD1

PLA2G10	PLA2G12A	PLA2G12B	PLA2G16	PLA2G1B	PLA2G2A	PLA2G2C	PLA2G2D	PLA2G2E	PLA2G2F
PLA2G3	PLA2G4A	PLA2G4B	PLA2G4C	PLA2G4D	PLA2G4E	PLA2G4F	PLA2G5	PLA2G6	PLA2G7
PLA2R1	PLAT	PLAU	PLB1	PLBD1	PLCG2	PLD1	PLD2	PLG	PLIN3
PLK1	PLK2	PLK3	PLK4	PLK5	PLP1	PLPPR4	PLSCR1	PLXNB1	PMAIP1
PMEL	PML	PMP2	PNPLA3	PNPLA8	POLA1	POLA2	POLB	POLG	Polr1d
POLR2A	POLR2B	POLR2C	POLR2D	POLR2E	POLR2F	POLR2G	POLR2H	POLR2I	POLR2J
POLR2K	Polr2l	POLR3A	POLR3B	POLR3C	POLR3D	POLR3F	POLR3G	PON1	POR
POU2AF1	POU2F2	POU3F1	PPARA	PPARD	PPARG	PPARGC1A	PPAT	PPBP	PPIA
PPIB	PPIC	PPID	PPM1B	PPP1R14C	PPP1R14D	PPP1R15A	PPP1R8	PPP3CA	PPP3CB
PPP3CC	PPP3R1	PPP3R2	PPP4C	PPT1	PQBP1	PRDM1	PRDM2	PRDX1	PRDX2
PRF1	PRIM1	PRIM2	PRKAA1	PRKAA2	PRKAB1	PRKAB2	PRKACA	PRKACB	PRKACG
PRKAG1	PRKAG2	PRKAR1A	PRKAR1B	PRKAR2A	PRKAR2B	PRKCA	PRKCB	PRKCD	PRKCE
PRKCG	PRKCH	PRKCI	PRKCQ	PRKCZ	PRKD1	PRKD3	PRKN	PRKRA	PRL
PRLH	PRMT3	PRNP	PROC	PROK1	PROKR2	PROX1	PRPF8	PRPS1	PRSS27
PRSS35	PRTN3	PSAP	PSEN1	PSEN2	PSENEN	PSIP1	PSMA1	PSMA2	PSMD14
PSMD2	PSTPIP1	PTAFR	PTEN	PTGDR2	PTGER1	PTGER2	PTGER4	PTGES	PTGS2
PTK2	PTK2B	PTPMT1	PTPN1	PTPN11	PTPN22	PTPN6	PTPRC	PTPRJ	PTPRN
PTPRZ1	PTX3	PVALB	PXYLP1	PYCARD	PYDC1	RAB11A	RAB11B	RAB11FIP1	RAB29
RAB33B	RAB6B	RAB9A	RABEP1	RABEPK	RACGAP1	RAD23B	RAD52	RAE1	RAF1
RAG1	RAG2	RANBP2	RAP1A	RAP1B	RAPGEF3	RARRES3	RASGRP1	RB1CC1	RBBP4
RBBP7	RBM42	RBP4	RBPJ	REL	RELA	RELB	REN	RET	RETN
RFFL	RFTN1	RFX5	RFXANK	RFXAP	RGMA	RHBDD3	RHOA	RHOB	RHOC
RHOD	RHOF	RHOG	RHOH	RHOJ	RHOQ	RHOT1	RHOT2	RHOU	RHOV
RICTOR	RIOK1	RIOK3	RIPK1	RIPK2	RNASE1	RNASE2	RNASE3	RNASE4	RNASE6
RNASE7	RNASE8	RNASEL	RND2	RND3	RNF111	RNF128	RNF150	RNF216	ROCK1
ROCK2	RORA	RORC	RPL13A	RPL18	RPL3	RPL35	RPL5	RPS10	RPS14
RPS15A	RPS16	RPS27A	RPS5	RPS6	RPS6KB1	RPSA	RRAGC	RRAS	RRM1
RRM2	RSAD2	RTP4	RTRAF	RUNX1	RUVBL2	RXRA	RXRB	RXRG	S100A12
S100A8	S100A9	S1PR1	SAA1	SAA4	SACS	SAE1	SAFB	SAMD9	SAMHD1
SAMSN1	SAP130	SAP18	SAP30	SAP30L	SASH3	SATB1	SCARB1	SCG2	SCGB1A1
SCGB3A1	SCN1A	SCN1B	SCN5A	SDC1	SDHA	SDHAF1	SDHAF4	SDHB	SDHD
SEC14L2	SELE	SELENOK	SELL	SELP	SELPLG	SEMA4A	SEMA4D	SERINC3	SERINC5
SERPINA1	SERPINA10	SERPINA12	SERPINA3	SERPINA4	SERPINA5	SERPINA6	SERPINA7	SERPINA9	SERPINB9
SERPINE1	SERPING1	SETD2	SF3A1	SF3B1	SF3B6	SFPQ	SFTPA1	SFTPB	SFTPD
SGCA	SGK1	SGPL1	SH2D1A	SH2D1B	SHC1	SHCBP1	SHMT1	SIAH1	SIGIRR
SIGLEC1	SIGLEC10	SIGMAR1	SIKE1	SIN3A	SIN3B	SIRPG	SIRT1	SIT1	SLA2
SLAMF1	SLAMF6	SLC11A1	SLC19A1	SLC22A2	SLC22A6	SLC25A1	SLC33A1	SLC6A19	SLC6A3
SLC6A4	SLC9A3R1	SLFN11	SLFN12	SLFN13	SLIT2	SLPI	SLURP1	SLX4	SMAD1
SMAD2	SMAD3	SMAD5	SMARCA4	SMARCB1	SMPD1	SMU1	SMURF1	SMYD3	SNAPIN
SNCA	SNRNP70	SNRPF	SNW1	SNX10	SNX6	SNX9	SOCS1	SOCS2	SOCS3
SOCS4	SOCS5	SOCS6	SOCS7	SOD1	SON	SOS1	SOS2	SOX5	SP100
SP110	SPACA3	SPAM1	SPHK1	SPHK2	SPI1	SPN	SPNS2	SPON2	SPP1
SPRED2	SPRED3	SPRY1	SPRY2	SPRY4	SQSTM1	SRC	SREBF2	SRPK1	SRPK2

SRRM2	SRSF1	SSRP1	SST	ST6GAL1	STAB1	STAT1	STAT2	STAT3	STAT4
STAT5A	STAT5B	STAT6	STIM1	STIM2	STK11	STK17B	STK39	STK4	STMN1
STX10	STX11	STX17	STX5	STXBP2	SUCNR1	SULF2	SUMO1	SUMO2	SUMO3
SUMO4	SUPT16H	SUPT6H	SUZ12	SVIL	SWAP70	SYK	TAB1	TAB2	TAB3
TAC1	TACR1	TACSTD2	TAF13	TAGAP	TAMM41	TANK	TAOK1	TAOK2	TAP1
TARDBP	TARM1	TBC1D10C	TBK1	TBL1XR1	TBL3	TBP	TBX21	TCF3	TCL1A
TCN1	TCP1	TEC	TERF2	TFE3	TFF2	TFRC	TG	TGFA	TGFB1
TGFB2	TGFB3	TGFBR1	TGFBR2	TGFBR3	THBS1	THPO	THRA	THRB	THY1
TICAM1	TICAM2	TIGIT	TIMP1	TIRAP	TK1	TK2	TLR1	TLR10	TLR2
TLR3	TLR4	TLR5	TLR6	TLR7	TLR8	TLR9	TMEM173	TMPO	TMPRSS11D
TMPRSS2	TNC	TNF	TNFAIP3	TNFAIP8L2	TNFRSF10A	TNFRSF10B	TNFRSF11B	TNFRSF12A	TNFRSF13B
TNFRSF13C	TNFRSF14	TNFRSF18	TNFRSF1A	TNFRSF1B	TNFRSF21	TNFRSF4	TNFRSF6B	TNFRSF9	TNFSF10
TNFSF11	TNFSF12	TNFSF13	TNFSF13B	TNFSF14	TNFSF15	TNFSF18	TNFSF4	TNFSF8	TNFSF9
TNIP1	TNIP2	TNK2	TNPO1	TNPO3	TNS3	TOB1	TOLLIP	TOMM40	TOP2A
TOP2B	TP53	TP53BP2	TPO	TPT1	TRADD	TRAF1	TRAF2	TRAF3	TRAF3IP1
TRAF3IP2	TRAF4	TRAF5	TRAF6	TRAP1	TREM1	TREM2	TREML2	TREML4	TRERF1
TREX1	TRIM10	TRIM11	TRIM13	TRIM14	TRIM15	TRIM21	TRIM22	TRIM23	TRIM24
TRIM25	TRIM26	TRIM27	TRIM28	TRIM31	TRIM32	TRIM35	TRIM36	TRIM37	TRIM38
TRIM42	TRIM44	TRIM45	TRIM47	TRIM5	TRIM50	TRIM55	TRIM56	TRIM58	TRIM59
TRIM6	TRIM60	TRIM61	TRIM62	TRIM63	TRIM65	TRIM66	TRIM67	TRIM7	TRIM71
TRIM8	TRIM9	TRIO	TRIP6	TRMT61A	TRPM4	TRPV2	TSG101	TSLP	TSPAN2
TSPOAP1	TSSK6	TUBA1A	TUBA1C	TUBA4A	TUBA8	TUBB	TUBB1	TUBB2A	TUBB3
TUBB4A	TUBB4B	TUBD1	TUBE1	TUBG1	TUBG2	TWIST1	TWSG1	TXK	TXLNA
TXN	TXNIP	TXNL4A	TYK2	TYMP	TYMS	TYR	TYRO3	TYROBP	UBA52
UBA7	UBAC2	UBASH3A	UBASH3B	UBB	UBC	UBD	UBE2E2	UBE2I	UBE2L6
UBE2N	UBE3C	UBP1	UBQLN4	UGCG	UGT1A1	UGT1A4	ULBP1	ULBP2	ULBP3
ULK1	UNC13D	UNC93B1	UPP1	URI1	USE1	USP13	USP15	USP18	USP2
USP21	USP25	USP6	USP7	VAMP8	VANGL1	VAPB	VAV1	VAV2	VAV3
VCAM1	VCP	VDR	VEGFA	VEGFB	VEGFC	VEGFD	VIM	VIP	VIPR1
VIPR2	VNN1	VNN2	VPS11	VPS16	VPS18	VPS33A	VPS33B	VPS39	VPS41
VPS4A	VPS4B	VSIR	VTCN1	VTI1B	VWF	WAS	WDFY3	WDR83	WIPF1
WIPI1	WNT1	WNT2	WNT3A	WNT4	WNT5A	WNT7A	WNT9A	WRN	XAB2
XBP1	XCL1	XCL2	XCR1	XIAP	XPC	XPNPEP1	XPO1	XPR1	XRCC1
XRCC2	XRCC3	XRCC4	XRN1	YBX1	YY1	ZAP70	ZBP1	ZBTB12	ZBTB14
ZBTB2	ZBTB7B	ZC3H12A	ZC3H12D	ZC3HAV1	ZDHHC17	ZEB1	ZEB2	ZFHX3	ZMYND11
ZNF280B	ZNF436	ZRANB1							

Table S2: 2483 genes are represented in the biological filter in the IVA filtering process. The genes originate from the following IVA gene lists: Interferon, Anti-inflammatory response, Viral replication (Replication of virus), Viral replication (Viral life cycle), Autophagy, CNS inflammation, Poliomyelitis, Poliomyelitis virus replication, Poliomyelitis virus release, Post-polio syndrome, Lymphocyte activation, Antiviral response, Innate immune response, Toll-like receptor signaling, Interferon response, RNA viruses activation, RNA viruses replication, Activation of IRF by cytosolic pattern recognition receptors, Role of pattern recognition receptors in recognition of bacteria and viruses, Pattern recognition receptor, NF-kB, Cytokine, Chemokine, Regulation by host of antiviral response, Proinflammatory response, Role of RIG1-like receptor in antiviral

innate immunity, or are associated with PV, innate immune sensing, signalling, or related to autophagy based on the literature.

Table S3: Additional information on the variants and variant-containing genes from the PPM cohort

ID	Gene Name	Transcript ID	Gene	RVIS (%)	GDI (%)	Mis. Z	LoF pLI	SIFT	dbSNP ID	GnomAd Frequency (%)
**1**	CREB-binding protein	NM_004380.2	*CREBBP*	0.217	59.91	5.58	1	T	368145743	0.034
	VPS16, CORVET/HOPS Core Subunit	NM_022575.3	*VPS16*	32.8	48.82	0.45	0	D	764631016	0.001
**2**	Coiled-coil and c2 domain containing 1a	NM_017721.4	*CC2D1A*	97.7	86.13	0.52	0	T	199644216	0.06
	Tumor protein p53 binding protein 2	NM_001031685.2	*TP53BP2*	5.73	66.28	1.09	0	D		
**3**	Endoplasmic reticulum aminopeptidase 1	NM_016442.4	*ERAP1*	71.9	98	-1.07	0	T		
	Myxovirus (influenza virus) resistance 1	NM_002462.4	*MX1*	20.2	92.22	-0.56	0	T	201917562	0.003
	TNFAIP3 interacting protein 1	NM_001252385.1	*TNIP1*	83.5	73.75	0.41	0.93	D	776324464	
**4**	DEAH-Box Helicase 36	NM_020865.2	*DHX36*	2.2	95.6	1.11	0.99	T	777343688	
	Unc-51 Like Autophagy Activating Kinase 1	NM_003565.2	*ULK1*	17	76.17	0.9	0.96			
**6**	Nitric oxide synthase 2, inducible	NM_000625.4	*NOS2*	17.2	89.64	1.41	0	D	377644483	0.003
	BCL2 Interacting Protein 2	NM_001320674.1	*BNIP2*	32.3	94.16	-1.53	0	T		
	cholinergic receptor nicotinic alpha 1 subunit	NM_000079.3	*CHRNA1*	23.8	53.82	0.64	0	D	147488907	0.045
**7**	Ubiquitin-like modifier activating enzyme 7	NM_003335.2	*UBA7*	69	61.64	0.48	0	D	149590583	0.01
	Cathepsin L	NM_001912.4	*CTSL*	49.5	28.21	-0.54	0.01	D	375548695	0.002
	Cathepsin C	NM_001814.5	*CTSC*	23.8	67.42	-0.84	0	D	146182103	0.013
	FBJ murine osteosarcoma viral oncogene homolog	NM_005252.3	*FOS*	62.5	22.85	1.52	0.44	D		
**8**	Annexin A6	NM_001155.4	*ANXA6*	74.6	42.6	-1.01	0	D	201366459	0.042
	Matrix metallopeptidase 8 (neutrophil collagenase)	NM_002424.2	*MMP8*	94	60.29	-1.29	0		141116762	0.067
**9**	Guanylate Binding Protein 1	NM_002053.2	*GBP1*	69.9	81.79	-0.85	0	D	142020556	0.009
	Cholinergic Receptor Nicotinic Alpha 7 Subunit	NM_000746.5	*CHRNA7*	57.6	25.89			T		0.002
	Ubiquitin Specific Peptidase 25	NM_001283041.2	*USP25*	6.9	54.96	0.7	0.99	D		0.001
**10**	Unc-51 Like Autophagy Activating Kinase 1	NM_003565.2	*ULK1*	17	76.17	0.9	0.96	D	532784108	
	Annexin A5	NM_001154.3	*ANXA5*	51.3	29.36	0.15	0	T		
	Rho GTPase activating protein 21	NM_020824.3	*ARHGAP21*	3.62	62.83	2.96	1	T		
**11**	BCL2 interacting protein 3	NM_004052.3	*BNIP3*	53.8	14.39	0.76	0.05	D	547824692	
**12**	TNF Receptor Associated Factor 2	NM_021138.3	*TRAF2*	12.7	28.34	1.86	1	D	142412558	0.017
	Protein tyrosine phosphatase, non-	NM_015967.6	*PTPN22*	86.5	55.89	-0.94	0	D	74163660	0.018

	receptor type 22 (lymphoid)									
	Nucleotide-binding oligomerization domain containing 1	NM_006092.3	*NOD1*	87.7	84.7	-0.12	0	D		
	Tumor protein p53 binding protein 2	NM_001031685.2	*TP53BP2*	5.73	66.28	1.09	0	D	138193509	0.012
**15**	Cholinergic Receptor Nicotinic Gamma Subunit	NM_005199.4	*CHRNG*	65.5	65.4	-0.32	0	D	16829216	0.008
	Phorbol-12-myristate-13-acetate-induced protein 1	NM_021127.2	*PMAIP1*	63.8	62.89	0	0.46	D		0.003
	VPS18, CORVET/HOPS Core Subunit	NM_020857.2	*VPS18*	8.72	52	2.01	0.94	D	755831113	0.009
**16**	ATG7 autophagy related 7 homolog (S. cerevisiae)	NM_006395.2	*ATG7*	26.2	59.69	0.79	0	D	200259863	0.02
	Cholinergic Receptor Nicotinic Alpha 5 Subunit	NM_000745.3	*CHRNA5*	67.7	79.21	0.32	0	T	56351164	0.018
**17**	Annexin A6	NM_001193544.1	*ANXA6*	74.6	42.6	-1.01	0	T		
	Tripartite Motif Containing 67	NM_001300889.1	*TRIM67*	34.4	41.64	2.79	0.67	T	369734440	0.002
**18**	Complement component 3a receptor 1	NM_004054.3	*C3AR1*	44.7	27.24	-1.55	0	D	765631714	0.002
	Cholinergic Receptor Nicotinic Alpha 10 Subunit	NM_020402.3	*CHRNA10*	92.3	64.23	-0.31	0		57724831	0.013
	Matrix metalloproteinase 2	NM_004530.5	*MMP2*	6.87	49.57	1.22	0.79	D	766384293	0.001

Table S3: Following identification of the variants by Ingenuity, the variants were evaluated for impact on protein level (SIFT and PolyPhen-2), and genetic intolerance of the variant containing genes (RVIS, GDI, LoF pLI and Missense Z). A SIFT score predicts whether an amino acid substitution affects protein function. The SIFT score ranges from 0.0 (deleterious/damaging) to 1.0 (tolerated/benign). The score can be interpreted as follows: 0.0 to 0.05: Variants with scores in this range are considered deleterious/damaging (D). 0.05 to 1.0: Variants with scores in this range are predicted to be tolerated (T). *RVIS:* Residual variance intolerance score. RVIS estimates the percentage of more intolerant genes according to data from NHLBI-ESP6500 data set. The score assesses whether genes have relatively more or less functional genetic variation than expected based on the apparently neutral variation found in the gene. *GDI:* Gene damaging index. GDI is the accumulated mutational damage of genes in the human population, hence GDI might be used to exclude variants found in highly damaged genes that are unlikely to be disease-causing. We used GDI and RVIS only as guidance in an analysis combining different tools; *Mis. Z:* Missense Z. ExAC missense Z positive score indicates an intolerant gene (fewer variants than expected), and a negative Z score indicates a gene with more variants than expected; *LoF pLI*: A score < 0.1 indicates a tolerant gene, a score >0.9 indicates a completely intolerant gene; *dbSNP ID*: Single nucleotide polymorphism database identification number. See Table 1 and Supplementary method 2 for more information of the variants and the variant-containing genes.

Table S4. Frequency of common single nucleotide polymorphisms associated with enterovirus infection

Gene	dbSNP ID	Risk allele (bold)	GnomAD Frequency (%)	Frequency in PPM cohort (%) (homo/hetero)	Reference
IFIH1	rs3747517	T>**C**	67.7	83.3 (12/6)	[44]
	rs35732034	A>**G**	0.6	0	[44]
	rs1990760	G>**A**	49.9	72.2 (10/6)	[45, 46]
TLR3	rs1914926	C>**G**	80.8	NS	[44]
	rs3775291	C>**T**	27.4	41.7 (3/9)	[47]
IFNAR1	rs2843710	C>**G**	37.4	NS	[48]

Table S4. Frequency of common single nucleotide polymorphisms (SNPs) associated with increased susceptibility to enterovirus infection
The frequencies of the two alleles in *IFIH1* (rs3747517 and rs1990760) as well as one allele in *TLR3* (rs3775291) were increased in the PPM cohort compared to GnomAD frequency. The common SNPs in TLR3 (rs1914926) and IFNAR1 (rs2843710) are located outside the exons, and therefore WES does not cover these regions. *Homo/hetero:* numbers of patient homozygous or heterozygous for the allele. *NS:* Not sequenced. *IFIH1*: Interferon induced with helicase C domain 1. *TLR3*: Toll-like receptor 3. *IFNAR1*: Interferon alpha and beta receptor subunit 1

Table S5: Variants identified in the HSE cohort

ABCB1	ABCC2	ABCC2	ABCC3	ABCC3	ABCC4	ACADVL	ACP5	AHNAK	ANXA5
APOB	ARHGAP33	ATF2	ATG2A	ATG2B	ATP1A2	ATP6V1A	BCL3	BIVM-ERCC5	BLNK
BMPR2	C3	C3AR1	C5	CACNA1C	CACNA1D	CACNA1S	CALR	CAMK4	CARD11
CBLL1	CCNT1	CDC42BPA	CHRM3	CHRM5	CHRND	CHRND	CHRND	CISH	CLSTN3
CLTCL1	CMTM4	COL2A1	COLEC12	CPT2	CRY1	CTBP1	CTBP2	CYP2D6	DHODH
DIABLO	DNAJC13	DNAJC5	DPP4	DTX2	DUOX2	EGR2	ENGASE	ENGASE	ENO1
EPAS1	EXOSC4	FGF7; FAM227B	FOXA2	FOXD1	FOXO3	GABRB2	GABRG3	GAD2	GAK
GAS6	GJA4	GOT2	GRIN2C	GRK5	GUCY1B3	HDAC4	HDAC5	HIST1H1B	HSH2D; RAB8A
HSP90B1	IFIT1	IKBKB	IL7R	INPP5D	IRS1	KIF1A	KRT16	LIG4	LIMK2
JUN	JUN	SIGLEC10	LRP1B	MAP3K12	MAP3K14	MAP3K8	MCL1	MFN1	MKNK1
MSH2	MTR	MVP	NCOA3	NDUFAF7; PRKD3	NOD2	NOTCH3	NOX3	NUP153	OAS1
ORAI1	PAX5	PCSK7	PELI1	PIK3C2G	PIK3R4	PIM2	JMJD7-PLA2G4B	PLB1	PLBD1
PLXNB1	PMEL	POLG	POLR2A; ZBTB4; SLC35G6	PPARA	PRKCD	PRKCD	PRKDC	PRKN	PSIP1
PSMD2	PXYLP1	RAP1A	RBPJ	RET	RHOT2	RNASEL	SACS	SACS	SACS
SIGLEC1	SLA2	SLC22A2	SMARCA4	SOCS5	SOS2	SPRED3	ST6GAL1	STAB1	STAT5A
STXBP2	SUPT16H	TACSTD2	TBK1	TFRC	TGFB1	TOP2A	TP53BP2	TRERF1	TRIM17; TRIM11
UNC13D	VPS33B	VPS4B	XRCC6	ZC3H12A	ZFHX3	ZFHX3			

Figure S1a and S1b: Innate immune responses in PBMCs

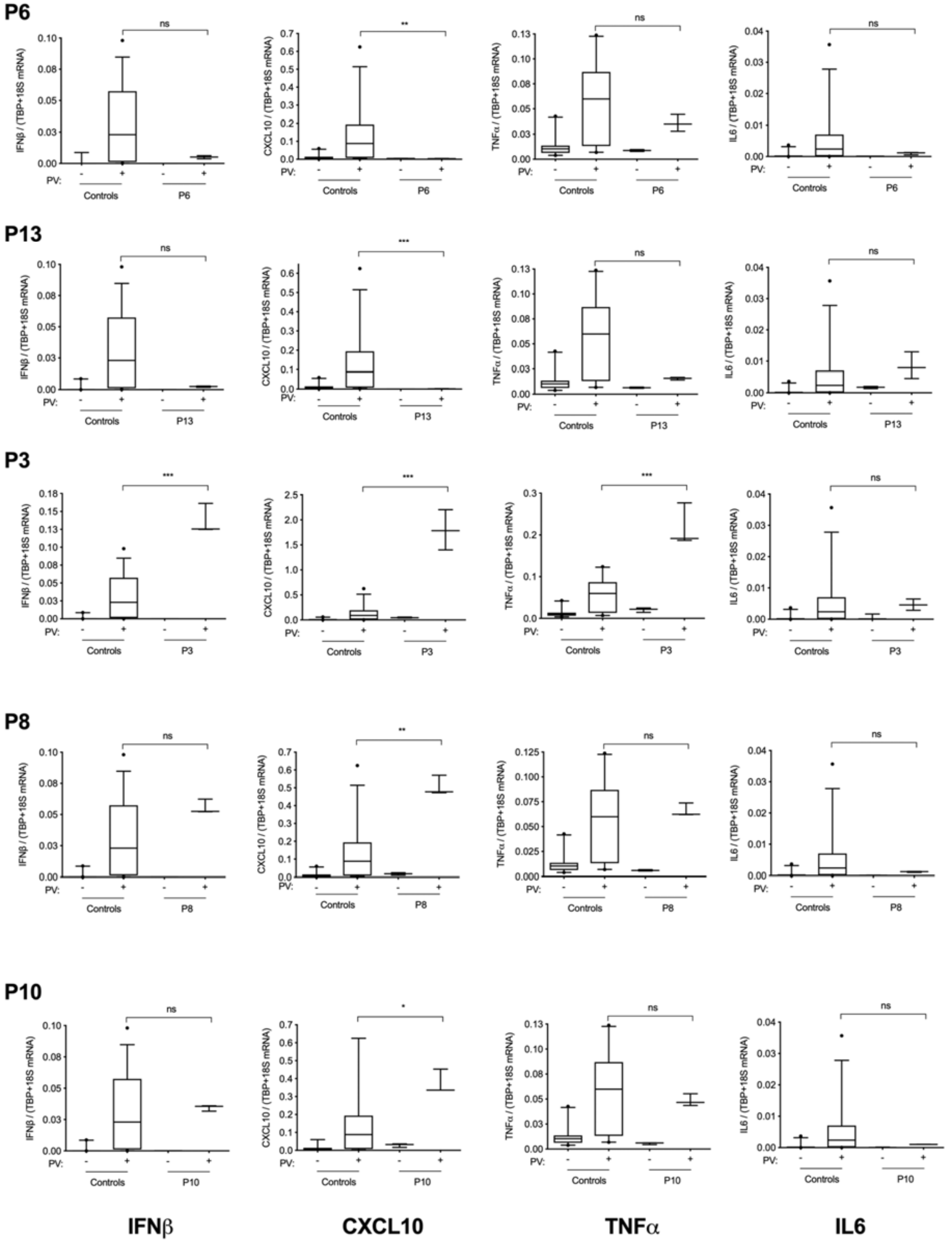

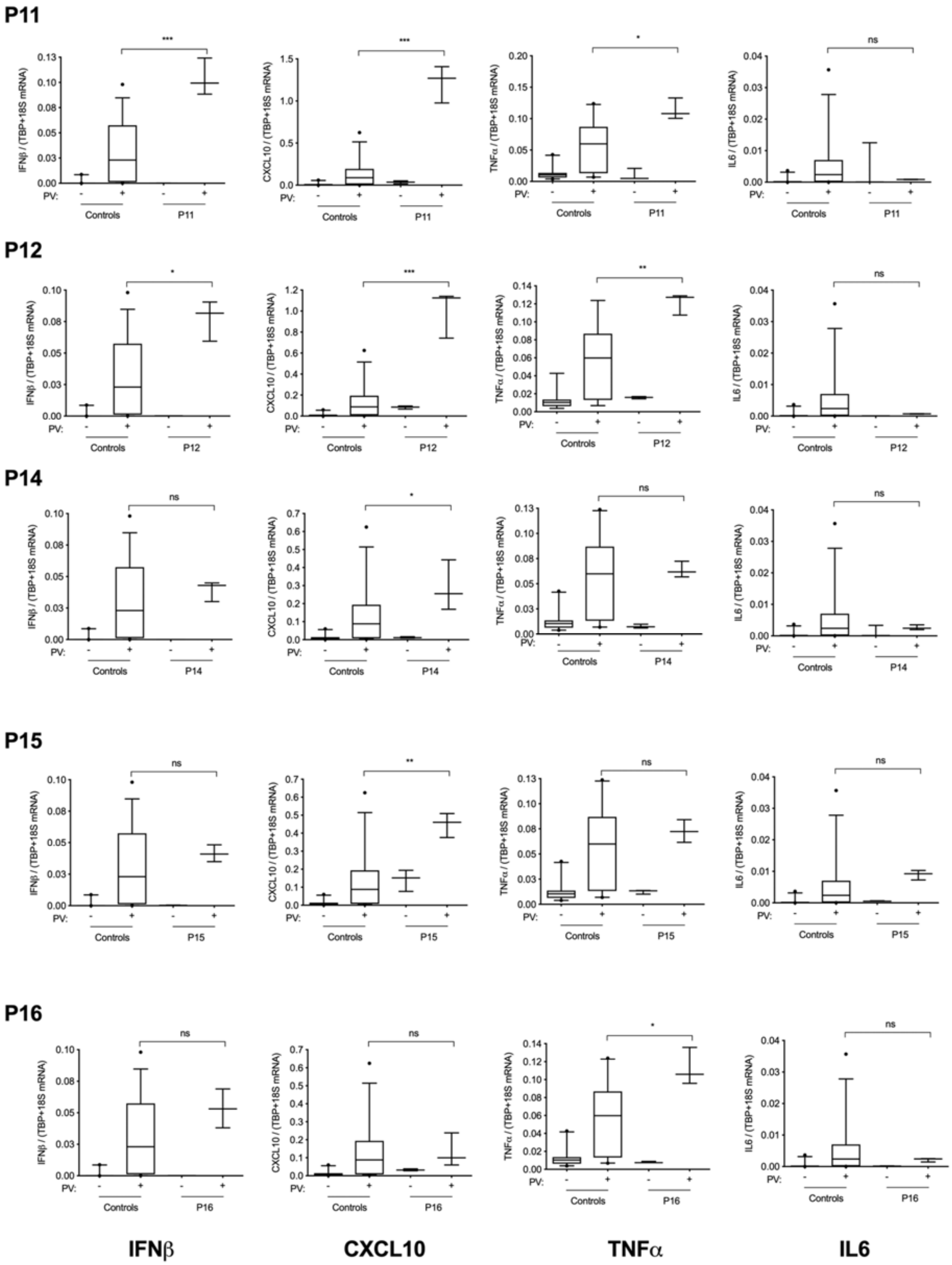
P11
P12
P14
P15
P16
IFNβ / (TBP+18S mRNA)
CXCL10 / (TBP+18S mRNA)
TNFα / (TBP+18S mRNA)
IL6 / (TBP+18S mRNA)
PV:
Controls
P11
P12
P14
P15
P16
***
**
*
ns
IFNβ
CXCL10
TNFα
IL6

Figure S1a and S1b: Innate immune responses in PBMCs

PBMCs from patients and controls were infected with poliovirus at MOI 100. Total RNA was harvested 6 hours following infection and subjected to RT-qPCR for measurement of IFNβ, CXCL10, TNFα, or IL6. Cytokine mRNA levels were normalized to the housekeeping gene TBP and compared with the pooled results of a total of nine controls. Patients are ordered by amount of CXCL10 production; P6 and P13 showed a significantly impaired CXCL10 response, whereas P3, P8, P10, P11, P12, P14, and P15 exhibited an elevated CXCL10 response to PV compared to controls. Moreover P3, P11, and P12 showed an increased IFNβ response and P3, P11, P12, and P16 showed an increased TNFα response. Data are not shown for patients without any significant differences in cytokine levels compared to controls. Data are shown as box plots with the 5–95% population, and outliers are shown as independent dots. Nonparametric Mann-Whitney ranked sum test was used for statistical analysis. *, $P \leq 0.05$; **, $P \leq 0.01$; ***, $P \leq 0.001$; ****, $P \leq 0.0001$. *PV;* poliovirus.

Figure S2: IFNβ response in MdMs

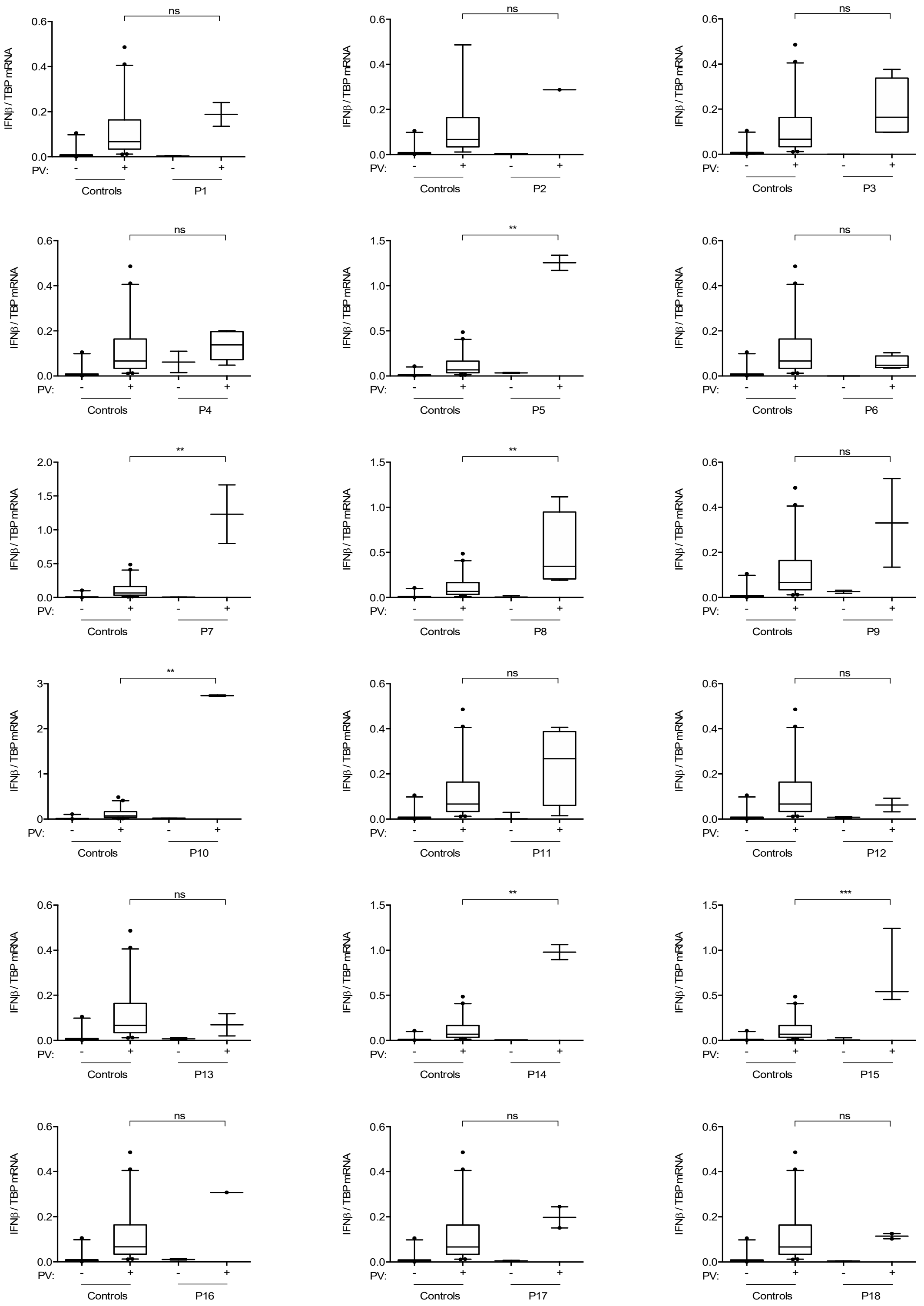

Figure S2: IFNβ response in MdMs

MdMs from patients and controls were infected with PV at MOI 10. Total RNA was harvested 6 hours following infection and subjected to RT-qPCR for measurement of IFNβ. Cytokine mRNA levels were normalized and compared with the pooled results of a total of fourteen controls. Data are shown as box plots with the 5–95% population, and outliers are shown as independent dots. Nonparametric Mann-Whitney ranked sum test was used for statistical analysis. *, $P \leq 0.05$; **, $P \leq 0.01$; ***, $P \leq 0.001$; ****, $P \leq 0.0001$. *PV;* poliovirus

Figure S3. Type I IFN quantification in PBMCs following poliovirus infection

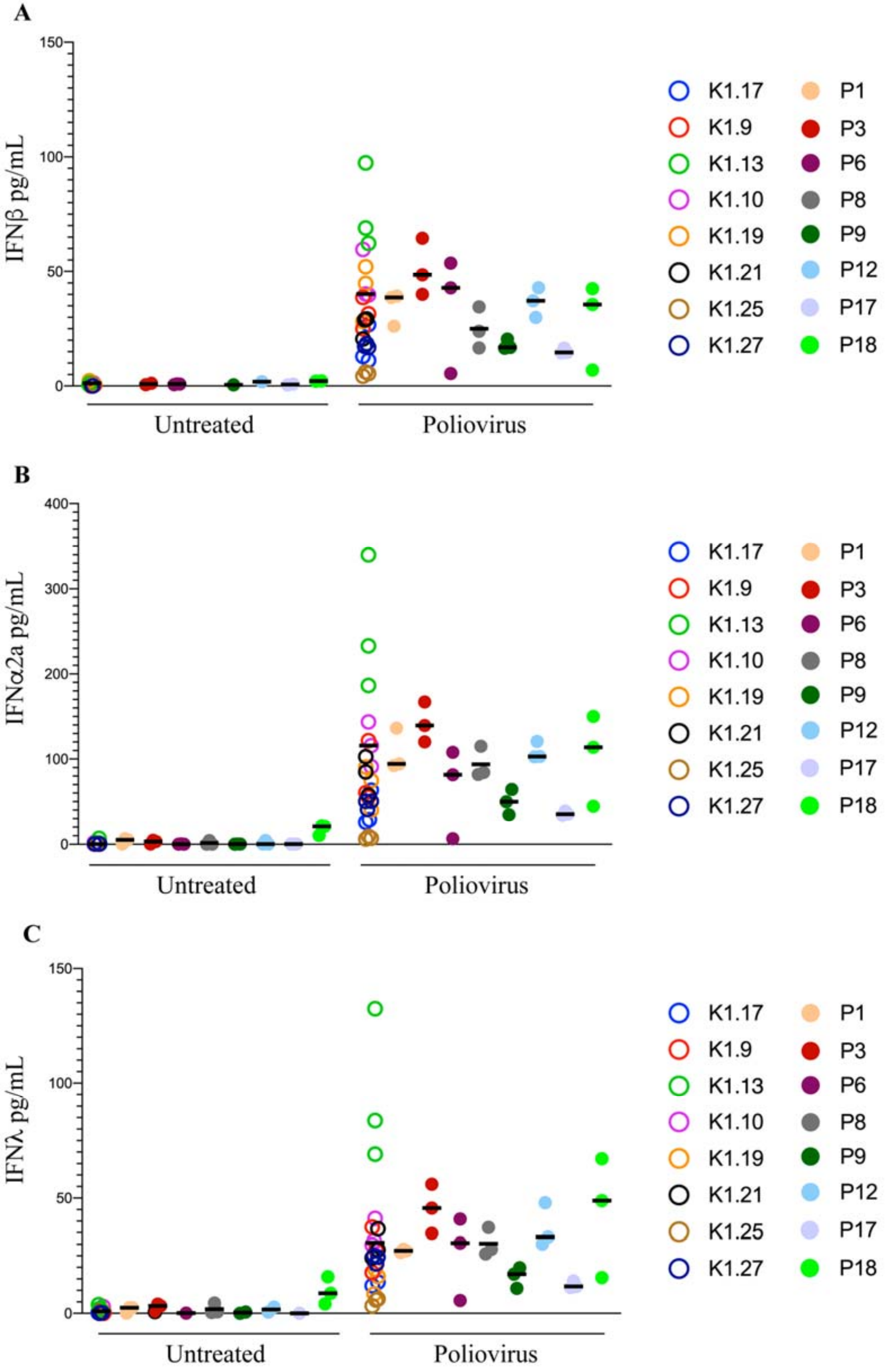

Figure S3. Type I IFN quantification in PBMCs following poliovirus infection

PBMCs from eight patients and eight controls were infected with poliovirus at an MOI of 100. Supernatants were harvested 24 hours following infection and subjected to mesoscale protein quantification of IFNβ, IFNα2a and IFNλ. The individual patients were compared with the pooled results of a total of eight controls. All values from poliovirus infection were above the limit of detection. Closed circles: Patients; open circles: Controls; bold lines indicate the median of biological triplicates; *HMW Poly(I:C):* High molecular weight polyinosinic-polycytidylic acid; *IFNβ*: Interferon beta; *IFNα2a*: Interferon alpha 2a; *IFNλ*: Interferon lambda. Nonparametric Mann-Whitney ranked sum test was used for statistical analysis. * $p \leq 0.05$; ** $p \leq 0.01$; *** $p \leq 0.001$; **** $p \leq 0.0001$

Figure S4. Type I IFN quantification in PBMCs following HMW Poly(I:C) stimulation

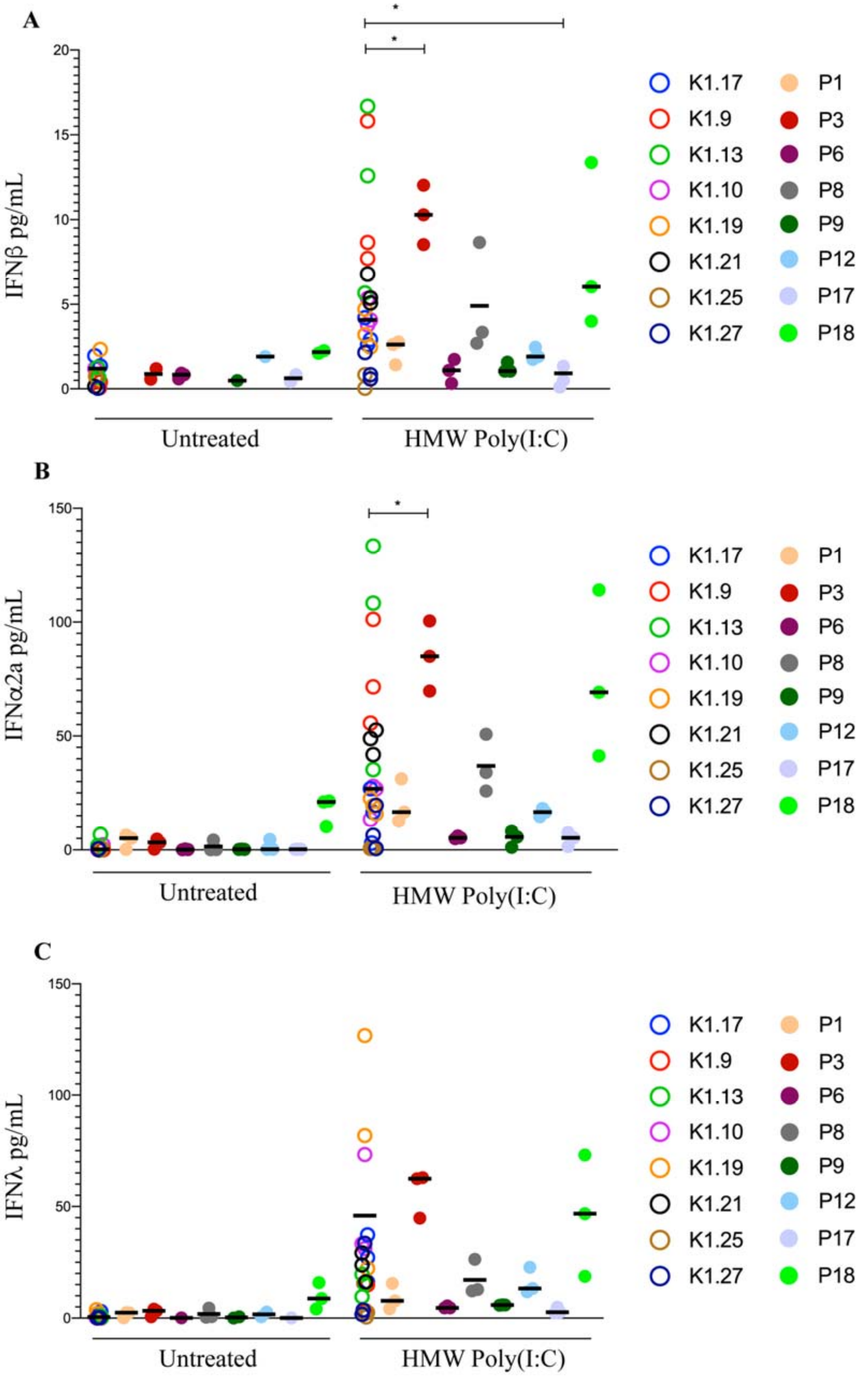

Figure S4. Type I IFN quantification in PBMCs following HMW Poly(I:C) stimulation

PBMCs from eight patients and eight controls were stimulated with 100 ug/mL HMW Poly(I:C). Supernatants were harvested 24 hours following infection and subjected to mesoscale protein quantification of IFNβ, IFNα2a and IFNλ. The individual patients were compared with the pooled results of a total of eight controls. All values from HWM Poly(I:C) stimulation were above the limit of detection. Closed circles: Patients; open circles: Controls; bold lines indicate the median of biological triplicates; *HMW Poly(I:C):* High molecular weight polyinosinic-polycytidylic acid; *IFNβ:* Interferon beta; *IFNα2*: Interferon alpha 2a; *IFNλ*: Interferon lambda. Nonparametric Mann-Whitney ranked sum test was used for statistical analysis.

References

1. Richards S, Aziz N, Bale S, Bick D, Das S, Gastier-Foster J et al. Standards and guidelines for the interpretation of sequence variants: a joint consensus recommendation of the American College of Medical Genetics and Genomics and the Association for Molecular Pathology. Genetics in medicine : official journal of the American College of Medical Genetics. 2015;17(5):405-24. doi:10.1038/gim.2015.30 [doi].

2. Vegna S, Gregoire D, Moreau M, Lassus P, Durantel D, Assenat E et al. NOD1 Participates in the Innate Immune Response Triggered by Hepatitis C Virus Polymerase. J Virol. 2016;90(13):6022-35. doi:10.1128/JVI.03230-15.

3. Zhang Z, Kim T, Bao M, Facchinetti V, Jung SY, Ghaffari AA et al. DDX1, DDX21, and DHX36 helicases form a complex with the adaptor molecule TRIF to sense dsRNA in dendritic cells. Immunity. 2011;34(6):866-78. doi:10.1016/j.immuni.2011.03.027 [doi].

4. Chang CH, Lai Lc Fau - Cheng H-C, Cheng Hc Fau - Chen K-R, Chen Kr Fau - Syue Y-Z, Syue Yz Fau - Lu H-C, Lu Hc Fau - Lin W-Y et al. TBK1-associated protein in endolysosomes (TAPE) is an innate immune regulator modulating the TLR3 and TLR4 signaling pathways. (1083-351X (Electronic)).

5. Lin D, Zhang M, Zhang MX, Ren Y, Jin J, Zhao Q et al. Induction of USP25 by viral infection promotes innate antiviral responses by mediating the stabilization of TRAF3 and TRAF6. Proc Natl Acad Sci U S A. 2015;112(36):11324-9. doi:10.1073/pnas.1509968112.

6. Wang Y, Shaked I, Stanford SM, Zhou W, Curtsinger JM, Mikulski Z et al. The autoimmunity-associated gene PTPN22 potentiates toll-like receptor-driven, type 1 interferon-dependent immunity. Immunity. 2013;39(1):111-22. doi:10.1016/j.immuni.2013.06.013.

7. Boyer NP, Monkiewicz C, Menon S, Moy SS, Gupton SL. Mammalian TRIM67 Functions in Brain Development and Behavior. eNeuro. 2018;5(3). doi:10.1523/ENEURO.0186-18.2018.

8. Olarerin-George AO, Anton L, Hwang YC, Elovitz MA, Hogenesch JB. A functional genomics screen for microRNA regulators of NF-kappaB signaling. BMC Biol. 2013;11:19. doi:10.1186/1741-7007-11-19.

9. Fang R, Jiang Q, Zhou X, Wang C, Guan Y, Tao J et al. MAVS activates TBK1 and IKKepsilon through TRAFs in NEMO dependent and independent manner. PLoS Pathog. 2017;13(11):e1006720. doi:10.1371/journal.ppat.1006720.

10. Chattopadhyay S, Yamashita M, Zhang Y, Sen GC. The IRF-3/Bax-mediated apoptotic pathway, activated by viral cytoplasmic RNA and DNA, inhibits virus replication. J Virol. 2011;85(8):3708-16. doi:10.1128/JVI.02133-10.

11. Fukushi M, Dixon J, Kimura T, Tsurutani N, Dixon MJ, Yamamoto N. Identification and cloning of a novel cellular protein Naf1, Nef-associated factor 1, that increases cell surface CD4 expression. FEBS Lett. 1999;442(1):83-8.

12. Vince JE, Pantaki D, Feltham R, Mace PD, Cordier SM, Schmukle AC et al. TRAF2 must bind to cellular inhibitors of apoptosis for tumor necrosis factor (tnf) to efficiently activate nf-{kappa}b and to prevent tnf-induced apoptosis. J Biol Chem. 2009;284(51):35906-15. doi:10.1074/jbc.M109.072256.

13. Yang H, Ma G, Lin CH, Orr M, Wathelet MG. Mechanism for transcriptional synergy between interferon regulatory factor (IRF)-3 and IRF-7 in activation of the interferon-beta gene promoter. Eur J Biochem. 2004;271(18):3693-703. doi:10.1111/j.1432-1033.2004.04310.x.

14. Zhu Z, Shi Z Fau - Yan W, Yan W Fau - Wei J, Wei J Fau - Shao D, Shao D Fau - Deng X, Deng X Fau - Wang S et al. Nonstructural protein 1 of influenza A virus interacts with human guanylate-binding protein 1 to antagonize antiviral activity. (1932-6203 (Electronic)).

15. Noda S, Tanaka K, Sawamura S, Sasaki M, Matsumoto T, Mikami K et al. Role of nitric oxide synthase type 2 in acute infection with murine cytomegalovirus. J Immunol. 2001;166(5):3533-41.

16. Morales DJ, Lenschow DJ. The antiviral activities of ISG15. J Mol Biol. 2013;425(24):4995-5008. doi:10.1016/j.jmb.2013.09.041.

17. Callahan MK, Popernack PM, Tsutsui S, Truong L, Schlegel RA, Henderson AJ. Phosphatidylserine on HIV envelope is a cofactor for infection of monocytic cells. J Immunol. 2003;170(9):4840-5.

18. Ma H, Kien F, Maniere M, Zhang Y, Lagarde N, Tse KS et al. Human annexin A6 interacts with influenza a virus protein M2 and negatively modulates infection. J Virol. 2012;86(3):1789-801. doi:10.1128/JVI.06003-11.

19. Zou Z, Meng Z, Ma C, Liang D, Sun R, Lan K. Guanylate-Binding Protein 1 Inhibits Nuclear Delivery of Kaposi's Sarcoma-Associated Herpesvirus Virions by Disrupting Formation of Actin Filament. J Virol. 2017;91(16). doi:10.1128/JVI.00632-17.

20. Zou Z, Meng Z, Ma C, Liang D, Sun R, Lan K. Guanylate-Binding Protein 1 Inhibits Nuclear Delivery of Kaposi's Sarcoma-Associated Herpesvirus Virions by Disrupting Formation of Actin Filament. LID - e00632-17 [pii] LID - 10.1128/JVI.00632-17 [doi]. (1098-5514 (Electronic)).

21. Wang S, Li H, Chen Y, Wei H, Gao GF, Liu H et al. Transport of influenza virus neuraminidase (NA) to host cell surface is regulated by ARHGAP21 and Cdc42 proteins. J Biol Chem. 2012;287(13):9804-16. doi:10.1074/jbc.M111.312959.

22. Jiang P, Nishimura T, Sakamaki Y, Itakura E, Hatta T, Natsume T et al. The HOPS complex mediates autophagosome-lysosome fusion through interaction with syntaxin 17. Mol Biol Cell. 2014;25(8):1327-37. doi:10.1091/mbc.E13-08-0447.

23. Itakura E, Kishi-Itakura C, Mizushima N. The hairpin-type tail-anchored SNARE syntaxin 17 targets to autophagosomes for fusion with endosomes/lysosomes. Cell. 2012;151(6):1256-69. doi:10.1016/j.cell.2012.11.001.

24. Kaminskyy V, Zhivotovsky B. Proteases in autophagy. Biochim Biophys Acta. 2012;1824(1):44-50. doi:10.1016/j.bbapap.2011.05.013.

25. Khaket TP, Singh MP, Khan I, Bhardwaj M, Kang SC. Targeting of cathepsin C induces autophagic dysregulation that directs ER stress mediated cellular cytotoxicity in colorectal cancer cells. Cell Signal. 2018;46:92-102. doi:10.1016/j.cellsig.2018.02.017.

26. Li L, Gao L, Song Y, Qin ZH, Liang Z. Activated cathepsin L is associated with the switch from autophagy to apoptotic death of SH-SY5Y cells exposed to 6-hydroxydopamine. Biochem Biophys Res Commun. 2016;470(3):579-85. doi:10.1016/j.bbrc.2016.01.102.

27. Tolskaya EA, Romanova LI, Kolesnikova MS, Ivannikova TA, Smirnova EA, Raikhlin NT et al. Apoptosis-inducing and apoptosis-preventing functions of poliovirus. J Virol. 1995;69(2):1181-9.

28. Zhang HM, Cheung P, Yanagawa B, McManus BM, Yang DC. BNips: a group of pro-apoptotic proteins in the Bcl-2 family. Apoptosis. 2003;8(3):229-36.

29. Zhang HM, Yanagawa B, Cheung P, Luo H, Yuan J, Chau D et al. Nip21 gene expression reduces coxsackievirus B3 replication by promoting apoptotic cell death via a mitochondria-dependent pathway. Circ Res. 2002;90(12):1251-8.

30. Sun Y, Leaman DW. Involvement of Noxa in cellular apoptotic responses to interferon, double-stranded RNA, and virus infection. J Biol Chem. 2005;280(16):15561-8. doi:10.1074/jbc.M412630200.

31. Rosebeck S, Sudini K, Chen T, Leaman DW. Involvement of Noxa in mediating cellular ER stress responses to lytic virus infection. Virology. 2011;417(2):293-303. doi:10.1016/j.virol.2011.06.010.

32. Kalamida D, Poulas K, Avramopoulou V, Fostieri E, Lagoumintzis G, Lazaridis K et al. Muscle and neuronal nicotinic acetylcholine receptors. Structure, function and pathogenicity. FEBS J. 2007;274(15):3799-845. doi:10.1111/j.1742-4658.2007.05935.x.

33. Albuquerque EX, Pereira EF, Alkondon M, Rogers SW. Mammalian nicotinic acetylcholine receptors: from structure to function. Physiol Rev. 2009;89(1):73-120. doi:10.1152/physrev.00015.2008.

34. Berrettini W, Yuan X, Tozzi F, Song K, Francks C, Chilcoat H et al. Alpha-5/alpha-3 nicotinic receptor subunit alleles increase risk for heavy smoking. Mol Psychiatry. 2008;13(4):368-73. doi:10.1038/sj.mp.4002154.

35. Kawashima K, Fujii T, Moriwaki Y, Misawa H, Horiguchi K. Non-neuronal cholinergic system in regulation of immune function with a focus on alpha7 nAChRs. Int Immunopharmacol. 2015;29(1):127-34. doi:10.1016/j.intimp.2015.04.015.

36. Tracey KJ. Reflex control of immunity. Nat Rev Immunol. 2009;9(6):418-28. doi:10.1038/nri2566.

37. Hao J, Simard AR, Turner GH, Wu J, Whiteaker P, Lukas RJ et al. Attenuation of CNS inflammatory responses by nicotine involves alpha7 and non-alpha7 nicotinic receptors. Exp Neurol. 2011;227(1):110-9. doi:10.1016/j.expneurol.2010.09.020.

38. Lustig LR, Peng H, Hiel H, Yamamoto T, Fuchs PA. Molecular cloning and mapping of the human nicotinic acetylcholine receptor alpha10 (CHRNA10). Genomics. 2001;73(3):272-83. doi:10.1006/geno.2000.6503.

39. Peng H, Ferris RL, Matthews T, Hiel H, Lopez-Albaitero A, Lustig LR. Characterization of the human nicotinic acetylcholine receptor subunit alpha (alpha) 9 (CHRNA9) and alpha (alpha) 10 (CHRNA10) in lymphocytes. Life Sci. 2004;76(3):263-80. doi:10.1016/j.lfs.2004.05.031.

40. Lakhan SE, Kirchgessner A, Tepper D, Leonard A. Matrix metalloproteinases and blood-brain barrier disruption in acute ischemic stroke. Front Neurol. 2013;4:32. doi:10.3389/fneur.2013.00032.

41. Stoermer KA, Morrison TE. Complement and viral pathogenesis. Virology. 2011;411(2):362-73. doi:10.1016/j.virol.2010.12.045.

42. Matsumoto N, Satyam A, Geha M, Lapchak PH, Dalle Lucca JJ, Tsokos MG et al. C3a Enhances the Formation of Intestinal Organoids through C3aR1. Front Immunol. 2017;8:1046. doi:10.3389/fimmu.2017.01046.

43. Kim S, Lee S, Shin J, Kim Y, Evnouchidou I, Kim D et al. Human cytomegalovirus microRNA miR-US4-1 inhibits CD8(+) T cell responses by targeting the aminopeptidase ERAP1. Nat Immunol. 2011;12(10):984-91. doi:10.1038/ni.2097.

44. Witso E, Cinek O, Tapia G, Brorsson CA, Stene LC, Gjessing HK et al. Genetic Determinants of Enterovirus Infections: Polymorphisms in Type 1 Diabetes and Innate Immune Genes in the MIDIA Study. Viral Immunol. 2015;28(10):556-63. doi:10.1089/vim.2015.0067.

45. Cinek O, Tapia G, Witso E, Kramna L, Holkova K, Rasmussen T et al. Enterovirus RNA in peripheral blood may be associated with the variants of rs1990760, a common type 1 diabetes associated polymorphism in IFIH1. PLoS One. 2012;7(11):e48409. doi:10.1371/journal.pone.0048409.

46. Pang L, Gong X, Liu N, Xie G, Gao W, Kong G et al. A polymorphism in melanoma differentiation-associated gene 5 may be a risk factor for enterovirus 71 infection. Clin Microbiol Infect. 2014;20(10):O711-7. doi:10.1111/1469-0691.12618.

47. Gorbea C, Makar KA, Pauschinger M, Pratt G, Bersola JL, Varela J et al. A role for Toll-like receptor 3 variants in host susceptibility to enteroviral myocarditis and dilated cardiomyopathy. J Biol Chem. 2010;285(30):23208-23. doi:10.1074/jbc.M109.047464.

48. Zou R, Zhang G, Li S, Wang W, Yuan J, Li J et al. A functional polymorphism in IFNAR1 gene is associated with susceptibility and severity of HFMD with EV71 infection. Sci Rep. 2015;5:18541. doi:10.1038/srep18541.
